# Systematic analysis of migration factors by MigExpress identifies essential cell migration control genes in non‐small cell lung cancer

**DOI:** 10.1002/1878-0261.12973

**Published:** 2021-05-14

**Authors:** Jagriti Pal, Andrea C. Becker, Sonam Dhamija, Jeanette Seiler, Mahmoud Abdelkarim, Yogita Sharma, Jürgen Behr, Chen Meng, Christina Ludwig, Bernhard Kuster, Sven Diederichs

**Affiliations:** ^1^ Division of Cancer Research Department of Thoracic Surgery Medical Center ‐ University of Freiburg Faculty of Medicine University of Freiburg German Cancer Consortium (DKTK) ‐ Partner Site Freiburg Germany; ^2^ Division of RNA Biology & Cancer German Cancer Research Center (DKFZ) Heidelberg Germany; ^3^ CSIR Institute of Genomics and Integrative Biology New Delhi India; ^4^ Leibniz Institute for Food Systems Technical University of Munich Freising Germany; ^5^ Bavarian Center for Biomolecular Mass Spectrometry (BayBioMS) Technical University of Munich Freising Germany; ^6^ Chair of Proteomics and Bioanalytics DKTK Partner Site Munich Freising Germany

**Keywords:** cancer cell migration, gene expression profiling, metastasis, non‐small cell lung cancer, proteomics, quantitative migration analysis, transcriptomics

## Abstract

Cell migration is an essential process in health and in disease, including cancer metastasis. A comprehensive inventory of migration factors is nonetheless lacking—in part due to the difficulty in assessing migration using high‐throughput technologies. Hence, there are currently very few screens that systematically reveal factors controlling cell migration. Here, we introduce MigExpress as a platform for the ‘identification of Migration control genes by differential Expression’. MigExpress exploits the combination of in‐depth molecular profiling and the robust quantitative analysis of migration capacity in a broad panel of samples and identifies migration‐associated genes by their differential expression in slow‐ versus fast‐migrating cells. We applied MigExpress to investigate non‐small cell lung cancer (NSCLC), which is the most frequent cause of cancer mortality mainly due to metastasis. In 54 NSCLC cell lines, we comprehensively determined mRNA and protein expression. Correlating the transcriptome and proteome profiles with the quantified migration properties led to the discovery and validation of FLNC, DSE, CPA4, TUBB6, and BICC1 as migration control factors in NSCLC cells, which were also negatively correlated with patient survival. Notably, FLNC was the least expressed filamin in NSCLC, but the only one controlling cell migration and correlating with patient survival and metastatic disease stage. In our study, we present MigExpress as a new method for the systematic analysis of migration factors and provide a comprehensive resource of transcriptomic and proteomic data of NSCLC cell lines related to cell migration.

## Graphical abstract

Cell migration is an essential process in development and disease—prominently in cancer metastasis. We present MigExpress, a high‐throughput approach to systematically identify factors governing cell migration by combining migration quantification with molecular profiling. MigExpress on a panel of 54 NSCLC cell lines discovered differentially expressed mRNAs, circRNAs, and proteins between fast‐ and slow‐migrating cells, which controlled migration.

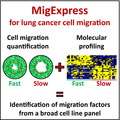

**Abbreviations**
BICC1: bicaudal C homolog 1
CDH1: E‐Cadherin
CDH2: N‐Cadherin
cDNA: complementary DNA
circRNA: circular RNA
CPA4: carboxypeptidase 4
DSE: dermatan sulfate epimerase
ECM: extracellular matrix
FLNA: filamin A
FLNB: filamin B
FLNC: filamin C
FPKM: fragments per kilobase per million
GO: Gene Ontology
LOXL2: lysyl oxidase‐like 2
LUAD: lung adenocarcinoma
MitC: mitomycin C
NSCLC: non‐small cell lung cancer
p53: tumor protein 53
RNA‐seq: RNA sequencing
RT‐qPCR: reverse transcription quantitative PCR
shRNA: short hairpin RNA
siRNA: small interfering RNA
TCGA: The Cancer Genome Atlas
TUBB6: tubulin beta 6
VIM: vimentin

## 1 Introduction

Cell migration plays a central role in a large number of developmental and physiological processes. During embryogenesis, cell migration is essential for gastrulation or development of the nervous system [1]. During adulthood, skin and intestinal cell layers are renewed when fresh epithelial cells migrate up from the basal layer and crypts, respectively [2]. Leukocytes migrate in response to infection and inflammation [3], while fibroblasts and vascular endothelial cells migrate to the site of wound healing [4]. Misregulation of cell migration can lead to serious pathological conditions such as congenital brain disorders, inflammatory and vascular diseases, and tumor metastasis [5].

Metastasis is a complex process in which cancer cells remodel the extracellular matrix (ECM), migrate into the vascular system, and move to distant sites where they establish metastatic nodules. Within a cell population, fast‐migrating cancer cells are likely to be more effective at giving rise to metastases than slower‐migrating cells [6]. Lung cancer is one tumor entity, especially prone to metastasis and the leading cause of cancer‐related death worldwide [7]. Over 80% of lung cancers are non‐small cell lung carcinomas (NSCLCs), which are further classified histologically into the major subtypes lung adenocarcinoma (LUAD) and squamous cell carcinoma [8]. More than 40% of NSCLC patients present already at diagnosis with the late‐stage IV cancer with distant metastases [9]. Additionally, vascular metastasis is often visible already in lower stages. Notably, fast‐migrating cancer cells contribute to significantly increased recurrence and reduced survival [10]. Despite many efforts to unravel coding and noncoding genes crucially involved in lung cancer migration, this process remains incompletely understood [11, 12, 13, 14].

While it is thus important to understand the molecular mechanisms that govern cancer cell migration, the determination of cell migration activity in higher throughput is a challenging task. Hence, while thousands of screens have been carried out for cancer cell viability and proliferation, only a handful of screens have identified genes controlling cell migration. For preselected genes, migration screens have been carried out by introducing cDNAs [15, 16, 17] or siRNAs/shRNAs [18, 19, 20, 21, 22, 23] into cancer cells. Only two broader shRNA screens were performed to identify factors that may affect migration in nontransformed human breast epithelial cells and in murine fibroblasts [24, 25]. In summary, only very few studies have tackled the challenge to systematically identify genes driving cell migration and are further limited by the number of genes, and the number of cell lines assessed or employed nontransformed cells.

In the present study, we present MigExpress (identification of Migration control genes by differential Expression) as an effective approach to uncover genes governing cell migration by simultaneously quantifying cell migration and gene expression. Application of MigExpress to a broad panel of NSCLC cell lines discovers and functionally validates a novel set of genes essential for lung cancer cell migration.

## 2 Materials and methods

### 2.1 Cell culture

All 54 NSCLC cell lines were cultured in RPMI medium (Gibco, Paisley, Renfrewshire, UK) supplemented with 10% FBS at 37 °C and 5% CO_2_ in a humidified incubator. Cells were obtained from ATCC (Gaithersburg, MD, USA) and / or fingerprint, and cells were regularly tested for mycoplasma.

### 2.2 Live cell staining and ORIS™ cell migration assay

Cells were incubated prior to seeding in medium with 2.5 µm DiR (1,1′‐dioctadecyl‐3,3,3′,3′‐tetramethylindotricarbocyanine iodide; D12731, molecular probes). 1 million cells were incubated in 1 mL for 15 min at 37 °C followed by a washing step.

For all 54 cell lines, cell migration was investigated using the Oris™ Cell Migration Assay TriCoated (Platypus Technologies). Cells were stained prior to seeding with a live dye (DiR) [26] and stained with DAPI at the end of the experiment. Cells were seeded on 96‐well Oris™ TriCoated plates (Platypus Technologies, Cat. No. SKU: CMATR1.101). Optimal seeding density for each cell line was determined beforehand. Plates were incubated for 18 h at 37 °C and 5% CO_2_ in a humidified incubator to allow cell attachment before the stopper was removed. The subsequent steps were performed according to the manufacturer’s instructions. Directly after removing the stopper, the 0‐h time point was recorded for all DiR‐stained cell lines (LI‐COR, Odyssey). After 24 and 48 h, this measurement was repeated. All plates were fixed after 48 h with 4% formaldehyde in PBS for 15 min at RT followed by DAPI staining with 300 nm DAPI (Thermo Fisher, Waltham, MA, USA—D1306) in PBS for 5 min. The mask was placed under the plate, and the stopper area was imaged using an Olympus Scan^R High Content Screening Station. The area was measured using imagej (NIH, Bethesda, MD, USA) and compared to a cohort of control wells determining the 0‐h time point (area of possible migration). Each cell line was tested at least in biological duplicates up to biological quintuplicates and technical duplicates up to technical quadruplicates.

### 2.3 RNA isolation

RNA was extracted using either the RNeasy Mini Kit (Qiagen, Hilden, Germany), the Direct‐zol™ RNA MiniPrep Kit (Zymo Research, Irvine, CA, USA), or the Quick‐RNA™ MiniPrep Kit (Zymo Research). Whole‐cell RNA that was used for RNA‐seq experiments was isolated using RNeasy Mini columns (Qiagen).

### 2.4 rRNA depletion and RNA‐seq analysis

The method has been described in detail before [27, 28]. In brief, rRNA depletion was performed on was performed on 5 μg RNA using the Ribo‐Zero Gold rRNA Removal Kit for human, mouse, and rat RNA as recommended by the manufacturer (Illumina, San Diego, CA, USA). Input RNA of 20 ng was subjected to RNA‐seq library preparation using the Sure Select Strand Specific RNA Library Prep for Illumina Multiplex Sequencing. 100‐bp paired‐end sequencing was performed on a HiSeq 4000 (Illumina). Sequencing quality metrics and rRNA contamination were determined with the EvalRSeq pipeline. Next, the reads were subjected to adapter removal and then uniquely mapped to the human genome GRCh38 using TopHat2 mapper allowing for up to two mismatches [29]. Gene expression was quantified in terms of FPKM values with ENSEMBL version 75 using easyRNASeq [30].

### 2.5 Mass spectrometry sample preparation and analysis

Cells at 80% confluency were scraped and lysed on ice in Tris/Urea lysis buffer (8 m Urea, 40 mm Tris/HCl pH 7.6, 1X protease inhibitor complete mini‐EDTA‐free (Hoffmann‐La Roche, Basel, Switzerland; 11836170001), 1× phosphatase inhibitors 1, 2, and 3 (Sigma‐Aldrich, Taufkirchen, Germany; P2850, P5726, P0044)). The protein concentration was determined by the bicinchoninic acid assay. The sample preparation for mass spectrometry included in‐solution tryptic digestion followed by solid‐phase extraction (SPE) peptide purification (SepPAK). Samples were tagged with TMT10plex™ reagent (Thermo Fisher Scientific™) [31], fractionated into 32 fractions using trimodal mixed‐mode chromatography [32], and subjected to mass spectrometric measurements using data‐dependent acquisition and multinotch MS3 mode [33] on a Thermo Scientific™ Fusion™ Lumos™ mass spectrometer (gradient length per fraction 1 h).

Data analysis was performed using MaxQuant (version 1.5.5.1) [34]. To normalize protein intensity distributions between all cell lines, 11 quantiles from 25% to 75% (by a step of 5%) were calculated for each cell line, respectively. These quantiles were aligned to the first sample based on a linear model; that is, intercept and slope were used to transform all protein intensities of the corresponding cell line. Next, we normalized the quantitative proteomic data across all TMT10 plex experiments using a common reference sample (pooled sample of three lung cancer cell lines: A549, NCI‐H460, and PC9) that was entailed in 2 out of 10 TMT channels in each TMT experiment. Based on the channel means of this reference, sample protein‐specific correction factors were computed for each TMT10plex experiment [35].

For the comparison of both RNA‐seq and mass spectrometry data between fast versus slow cell lines, the fold change was calculated for each coating (uncoated, collagen‐coated, and fibronectin‐coated) separately and the average fold change was used for candidate selection. For the determination of the statistical significance of differentially expressed genes, the fast versus slow cell lines were compared on the basis of their average migration capacity on the three coating surfaces.

### 2.6 Pathway analysis

The pathway analysis was carried out using DAVID analysis online tool (https://david.ncifcrf.gov) [36, 37]. The selected pathways are statistically significantly enriched with a minimum number of four genes.

### 2.7 siPOOL knockdown

For knockdown of the six targets using siPOOLs, cells were reverse‐transfected with 10 nm (final concentration) of nontargeting control (siNT) or siPOOL of all targets (siTOOLs Biotech) using 6 µL Lipofectamine™ RNAiMAX (Invitrogen, Waltham, MA, USA) in 6‐well plates, or 0.2 µL/well in 96‐well plates. For the scratch assays, cells were seeded at ~ 50% confluency in collagen I precoated 96‐well cell culture plates (IncuCyte^®^ ImageLock, 4379) and reverse‐transfected. Cells from 6‐well plates were used for RNA extraction. siPOOL sequences are listed in Table S1.

### 2.8 IncuCyte^®^ scratch assay

Scratch assays were performed using the WoundMaker™ (Essen Bioscience, Ann Arbor, MI, USA) and measured in the IncuCyte^®^ S3 Live‐Cell Analysis System according to the manufacturer’s instructions. Every 1 h, pictures of each well were taken. For the analysis of different cell lines in the correlation graph, the analysis was performed manually. The size of the wound was measured at three positions over each scratched area between 0 h and up to 24 h. The migrated area was calculated by the subtraction of the average size of the gap (measured at the three positions) after 24 h from the average size of the gap (measured at the three positions) at 0 h. Each cell line was tested in biological duplicates and technical triplicates/quadruplicates.

For scratch assays after siPOOL‐mediated knockdown, the plate was initially coated with collagen I rat tail (50 µg·mL^−1^). Cells were reverse‐transfected with siPOOLs and plated at 50% confluency. After 24 h, the scratch wound was made with WoundMaker™ (Essen Bioscience) and imaged in the IncuCyte^®^ S3 Live‐Cell Analysis System every 1 h for 24 h. For scratch assays in the presence of mitomycin C (Sigma‐Aldrich F1141), cells were reverse‐transfected in medium containing 10 µm MitC. After 24 h, the scratch was made and the wells were replenished with medium containing 7.5 µm MitC and images were recorded for 24 h at 1‐h intervals. All experiments were done in technical triplicates and biological triplicates to quintuplicates.

After recording the wound closure within the IncuCyte^®^ S3 Live‐Cell Analysis System, knockdown experiments were analyzed within the software. For all tested cell lines, the following setting was used: segmentation adjustment (0.7), hole fill (0.01), and adjust size (3). After analysis, information about wound confluence was exported for each well to every measured time point.

### 2.9 cDNA conversion and real‐time qPCR

For cDNA conversion, 2 µg of total RNA was subjected to reverse transcription using Thermo Scientific™ RevertAid RNA Transcriptase using random primers. For real‐time qPCR, equal amounts of cDNA were used with Applied Biosystems™ SYBR™ Green PCR Master Mix and Applied Biosystems™ StepOnePlus™. The sequences of all primers are provided in Table S2.

### 2.10 Proliferation assay

Ten thousand cells were plated in duplicates for each condition and each time point (48 and 72 h) in 24‐well plates. After 48 and 72 h, cells were trypsinized and resuspended in complete medium. An equal volume of trypan blue was mixed with the cells, and the cells were counted in a Bio‐Rad TC20™ cell counter. The initial cells plated were taken as cell count at 0 h.

### 2.11 circRNA analysis

Circular RNA expression had been previously analyzed in the RNA‐seq dataset of the 54 cell lines [28, 38]. Differentially expressed circRNAs, both at the gene level and at the back‐splice level, between fast‐ and slowly migrating cell lines were determined by calculating the fold change for each coating (uncoated, collagen‐coated, and fibronectin‐coated) separately, and the average fold change in all three coatings was used for candidate selection. Also, the statistical significance of the expression difference between fast‐ versus slowly migrating cell lines was calculated separately for each coating and the average significance value was used for selection. For the validation, outward‐facing (divergent) primers were used for RT‐qPCR. The primers are listed in Table S2.

## 3 Results

### 3.1 Robust quantification of cell migration with medium throughput

**Fig. 1:**
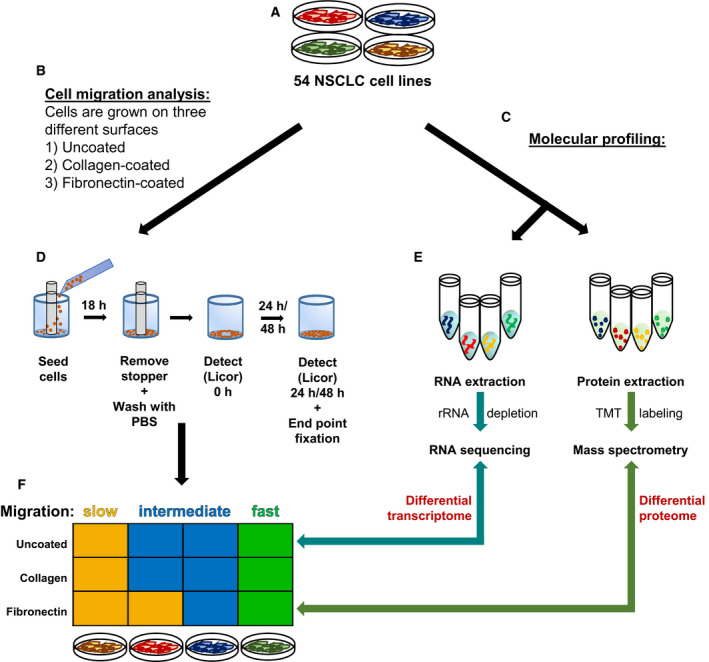
Schematic workflow of the MigExpress screen. (A) A panel of different cell lines is needed for a MigExpress screen—for this screen, 54 NSCLC cell lines were analyzed. (B) The cell migration capacity of each cell line was quantified on three different matrix surfaces—uncoated, collagen‐coated, and fibronectin‐coated. (C) Molecular profiling of 54 NSCLC cell lines. (D) ORIS™ assay. The cells were stained with DiR and seeded into a 96‐well plate with a stopper placed at the center. After 18 h, the stopper was removed and the cells were washed with PBS and detected as time point 0 h. After 24 and 48 h, the cells were washed and imaged by LI‐COR™. (E) The molecular profiling comprised RNA sequencing of rRNA‐depleted total RNA and mass spectrometry of TMT‐labeled proteins from whole‐cell lysates. (F) Cells were classified on the basis of their migration capacity into fast, medium, and slow cells. Differential gene expression and differential protein regulation were identified from RNA‐seq and mass spectrometry data between fast versus slow cell lines identified from the migration screen.

A panel of 54 NSCLC cell lines enriched for LUAD was screened for their migration capacities on different matrix surfaces (uncoated, collagen‐coated, and fibronectin‐coated) using the ORIS™ migration assay to quantitatively and robustly assess the migration capacity of each cell line. The cell lines were classified into ‘fast’, ‘medium’, and ‘slow’ cell lines depending on their migration properties. In parallel, the cell lines were subjected to RNA sequencing and mass spectrometric analyses for transcriptomic and proteomic profiling, respectively. Gene expression differences were determined from the RNA‐seq and mass spectrometry data to identify genes that were significantly up‐ or downregulated between the cells classified as fast or slow. The overall workflow of MigExpress is summarized in Fig. 1.

**Fig. 2:**
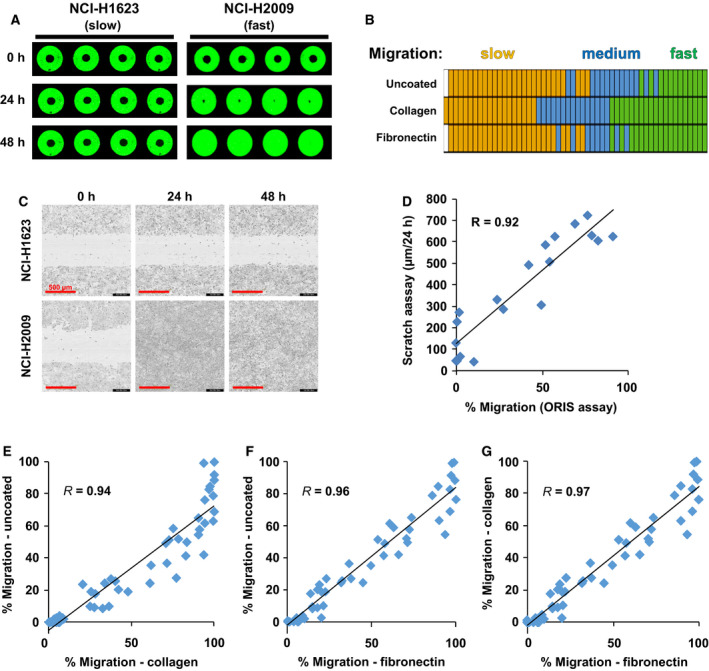
Quantification of migration capacity and validation. (A) Representative images of slow (NCI‐H1623) versus fast (NCI‐H2009) cell lines from ORIS™ assay. (B) Classification of 54 NSCLC cell lines by their migration capacity. Each row represents ORIS™ assay results on the respective matrix surfaces. Each column represents one cell line. Orange, blue, and green colors represent slow‐, medium‐, and fast‐migrating cells, respectively. White color represents experiment for which data were not available due to the inability of the cells to attach within 18 h on uncoated and fibronectin‐coated wells. Values of > 60%, > 75%, and > 60% for uncoated, collagen‐coated, and fibronectin‐coated matrix, respectively, were used for defining fast cells. Values of < 25% defined slow cells. (C) Representative images of slow (NCI‐H1623) versus fast (NCI‐H2009) cell lines from IncuCyte^®^ scratch assay for validation. The scale bar (red) indicates 500 µm. (D) Positive correlation between migration quantified by scratch assay versus by ORIS™ assay for 20 cell lines. (E‐G) Comparison of the percentage migration of each line on different matrix surfaces measured at 48 h. R represents Pearson’s correlation coefficient.

As an example, representative images of slow (NCI‐H1623) versus fast (NCI‐H2009) cell lines from the ORIS™ migration assay for 0, 24, and 48 h time points show an apparent difference in their migration capacities (Fig. 2A). In this assay, a stopper blocks cell growth in a defined area in the middle of the well. The migrating NCI‐H2009 cells quickly closed this gap within 24 h after removal of the stopper. In contrast, the NCI‐H1623 cells were unable to migrate into the central zone even within 48 h. A total of 18, 10, and 26 cell lines were found to be fast, medium, and slow on two or more of the above three matrix surfaces, respectively (Fig. 2B; Table S3). To confirm the robustness of the assay, we checked the variability among replicates by calculating the SEM for each cell line. The average SEM was < 5 for ORIS assays (using a scale from 0 to 100) on all three matrix surfaces (Fig. S1A).

Next, we further validated the platform for the quantitative analysis of cell migration. Cell proliferation is considered as a potentially confounding factor for the analysis of cell migration as cells proliferating faster could also enter uncovered areas faster. Hence, we also determined the proliferation rate of all 54 cell lines in terms of their cell doublings within 72 h. A correlation analysis of the proliferation rate and the migration capacity verified that there was no relevant correlation of cell proliferation with cell migration (*R *= −0.10; Fig. S1B). Hence, differences in proliferation rates did not affect the outcome of the migration quantitation.

To validate the ORIS™ assay results with an independent method, the migration capacity was validated using the IncuCyte^®^ scratch wound assay for a subset of 20 lung cancer cell lines. The results showed a highly significant correlation between the migration capacities determined by the two techniques with a correlation coefficient of *R* = 0.92 (Fig. 2C,D; Fig. S1C). In coherence with the ORIS™ assay, complete wound closure was observed for NCI‐H2009 cells in 24 h, while closure was negligible for the NCI‐H1623 cells even after 48 h (Fig. 2C).

Lastly, a high correlation (*R* = 0.94–0.97) was observed between the migration capacities of the NSCLC cells determined on different matrix surfaces (Fig. 2E‐G).

In summary, these data validate the ORIS™ as a robust platform to quantify cell migration for a broad panel of cell lines consistent with scratch assays and independent of cell proliferation.

### 3.2 Combining migration quantification with high‐throughput molecular profiling to identify genes essential for NSCLC cell migration

**Fig. 3:**
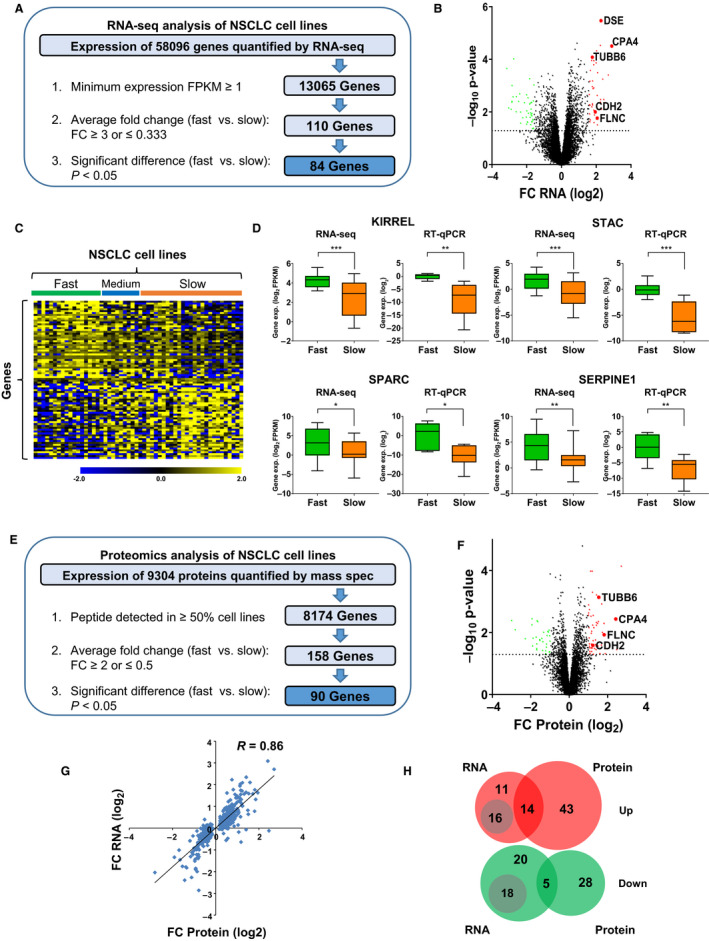
Transcriptomic and proteomic profiling of NSCLC cell lines. (A) Pipeline for the identification of candidate genes from RNA‐seq data. The analysis identified 84 candidate genes based on expression, regulation, and significance. (B) Volcano plot for 13065 genes that have a minimum expression of FPKM ≥ 1. The red dots represent significantly upregulated genes (fold change ≥ 3, *P* < 0.05), and the green dots represent significantly downregulated genes (fold change ≤ 0.333, *P* < 0.05). Selected candidates are indicated with larger circles. (C) Heat map of 84 candidate genes. Each row represents a gene, and each column represents a cell line. The first 18 columns are the fast‐migrating (green), the next 10 columns are the medium‐migrating (blue), and the last 26 cell lines are the slow‐migrating (orange) cell lines. In the heat map, yellow color indicates high, while blue color indicates low expression. (D) RT‐qPCR validation of selected upregulated candidates in eight fast versus eight slow cell lines. Boxplots: central line = median / box = 25% and 75% quartiles / whiskers = min and max. Statistical test: *t*‐test with Welch’s correction, *P*‐values: *P < 0.05, **P < 0.01, ***P < 0.001. (E) Pipeline for the identification of candidate genes from mass spectrometry data. The analysis identified 90 candidate genes based on expression, regulation, and significance. (F) Volcano plot for 8174 genes that have minimum expression with peptide detection in ≥ 50% of cell lines. The red dots represent significantly upregulated genes (fold change ≥ 2, *P* < 0.05), and the green dots represent significantly downregulated genes (fold change ≤ 0.5, *P* < 0.05). Selected candidates are indicated with larger circles. (G) Correlation between RNA‐seq and mass spectrometric data for the fold change in differentially expressed genes between fast versus slow cells (*n* = 525; genes with significant *P*‐value < 0.05 from both datasets, FPKM ≥ 1 in RNA‐seq data / detected in ≥ 50% cell lines in mass spec). R represents Pearson’s correlation coefficient. (H) Overlap between upregulated (red) and downregulated (green) candidates identified from RNA‐seq versus mass spectrometry data. The numbers in the gray circles represent genes that were regulated at the RNA level but not detected at the protein level. No overlap was observed in opposing directions of regulation among the candidate lists.

For transcriptome profiling, we performed strand‐specific RNA sequencing in replicates after depletion of ribosomal RNA to cover all types of RNA including nonpolyadenylated transcripts. The expression of 58 096 genes was quantified in the 54 cell lines (Fig. 3A, Table S4). To focus the analysis on robustly expressed genes, we selected 13065 genes, which were detected at a cutoff of minimum expression of FPKM ≥ 1. Of these, 110 genes had a fold change difference of more than threefold between fast versus slow cell lines. Finally, 84 of these genes reached statistical significance including eight noncoding RNAs and one pseudogene (Fig. 3A‐C, Table S5). To validate the differential expression patterns, we performed RT‐qPCR for 41 out of 84 genes, which had been selected for the strength of their regulation and the literature about their known functions, in a subpanel of eight fast versus eight slow cell lines. Notably, 38 out of 41 genes showed similar regulation in both RNA‐seq and RT‐qPCR data with 25 of them reaching statistical significance, underlining the reproducibility of the approach (Fig. 3D, Fig. S2A‐C, Table S6). Genes that are well‐known promoters (VIM and LOXL2 [39, 40]) or suppressors (CDH1 [41]) of NSCLC migration were also found to be up‐ or downregulated in fast compared with slow cells, respectively, further validating the MigExpress approach (Fig. S2D‐F).

Overall in the proteomic profiling of the 54 cell lines, a total of 9304 proteins were detected by mass spectrometry. Out of these, 8174 proteins fulfilled the criterion for a minimum of expression with a detectable signal in at least 50% of the cell lines (Table S7). Further selection based on a minimum fold change of twofold and *P*‐value < 0.05 between fast versus slow cell lines revealed 90 differentially expressed candidates based on proteomics (Fig. 3E‐F, Table S8).

Furthermore, we compared the regulation at the RNA and the protein level for 525 genes (with *P* < 0.05 for the RNA‐seq and mass spectrometry datasets and with minimum expression of FPKM ≥ 1 for RNA‐seq data and proteins detected in ≥ 50% cell lines for mass spectrometry data) and found a very high correlation (*R* = 0.86) for the fold change between fast versus slow cell lines between the transcript and the protein level (Fig. 3G).

When the independent candidate lists of 84 hits from transcriptomics and 90 hits from proteomics were compared, 34 genes were detected by RNA‐seq but not by mass spectrometry. For the remaining 50 hits from RNA‐seq, 19 genes (38%) displayed an overlap with the proteomic hits with 14 upregulated and five downregulated genes (Fig. 3H). Importantly, no overlaps were found for opposing directions of regulation between transcriptomics and proteomics. When the 43 proteins upregulated and the 28 proteins downregulated were analyzed manually at the RNA level without any filters, all 71 transcripts were regulated also concordantly in the same direction and none in the opposite direction, but then 14 were filtered out due to low expression at the RNA level and 57 did not reach threefold regulation. Gene Ontology (GO) pathway analysis of the upregulated genes merged from both candidate lists unraveled a significant enrichment of terms linked to migratory processes such as ECM organization, cell adhesion, or cell migration (Fig. S3A), while the 76 downregulated genes showed significant enrichment of specific metabolic processes (Fig. S3B).

In summary, MigExpress identified candidates linked to cell migration by combining differential migratory capacities of the broad panel of cell lines to their transcriptomic and proteomic profiles.

### 3.3 Candidates selected from MigExpress correlate with NSCLC patient survival

For the further characterization and validation of the hits obtained from our MigExpress screen in NSCLC, we selected six candidates upregulated in fast cells compared with slow cells: *CDH2* (N‐Cadherin) [42], which had already previously been prominently linked to cell migration served as positive control and was significantly upregulated in fast cells in the RNA‐seq and the mass spectrometry analysis. Among the genes not previously linked to cell migration according to GO, the following five candidates were selected for further validation: *CPA4* (carboxypeptidase 4 [43, 44]), *FLNC* (filamin C [45, 46]), and *TUBB6* (tubulin beta 6 [47]) as candidates with high fold change between fast versus slow cell lines in transcriptomic and proteomic data. Dermatan sulfate epimerase (*DSE* [48, 49]) had the second highest fold change difference from RNA‐seq data (after *CPA4*) and was not detected by mass spectrometry. *BICC1* (bicaudal C homolog 1 [50, 51]) was highly expressed in a subset of fast cell lines that represented LUAD. For all selected genes, we observed no significant correlation with the proliferation capacity of cells.

**Fig. 4:**
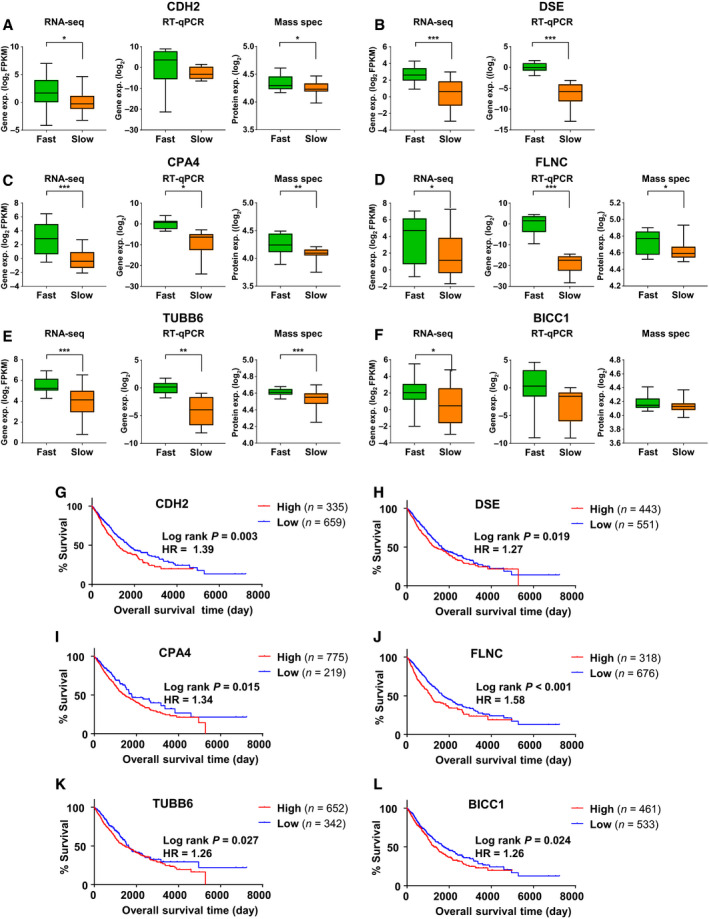
Gene expression and survival analyses of six candidate genes. (A‐F) Gene expression between fast versus slow cell lines from RNA‐seq (*n* = 54), RT‐qPCR (*n* = 16), and proteomic data (*n* = 54) for (A) *CDH2*, (B) *DSE*, (C) *CPA4*, (D) *FLNC*, (E) *TUBB6*, and (F) *BICC1*. DSE was not detected at the protein level. All six candidates exhibit upregulation in fast versus slow cells. Statistical test: *t*‐test with Welch’s correction, *P*‐values: *P < 0.05, **P < 0.01, ***P < 0.001. (G‐L) Kaplan–Meier survival plot of NSCLC patients (*n* = 994) with high versus low levels of the candidate genes—(G) *CDH2*, (H) *DSE*, (I) *CPA4*, (J) *FLNC*, (K) *TUBB6*, and (L) *BICC1*. Log‐rank test *P*‐values: *P < 0.05, **P < 0.01, ***P < 0.001. HR, hazard ratio.

All six selected genes were upregulated in the fast compared with the slow cell lines in our RNA‐seq, RT‐qPCR, and mass spectrometry data (Fig. 4A‐F). From The Cancer Genome Atlas (TCGA), we extracted expression and survival data for 994 NSCLC patients [52, 53]. Strikingly, the expression of all six selected genes was negatively correlated with the overall survival in this large NSCLC patient cohort with moderate hazard ratios but all reaching statistical significance. NSCLC patients with high expression of the individual genes showed significantly poorer survival compared with those expressing lower levels of the respective gene (Fig. 4G‐L).

### 3.4 Knockdown of candidate genes validates their importance in NSCLC cell migration

**Fig. 5:**
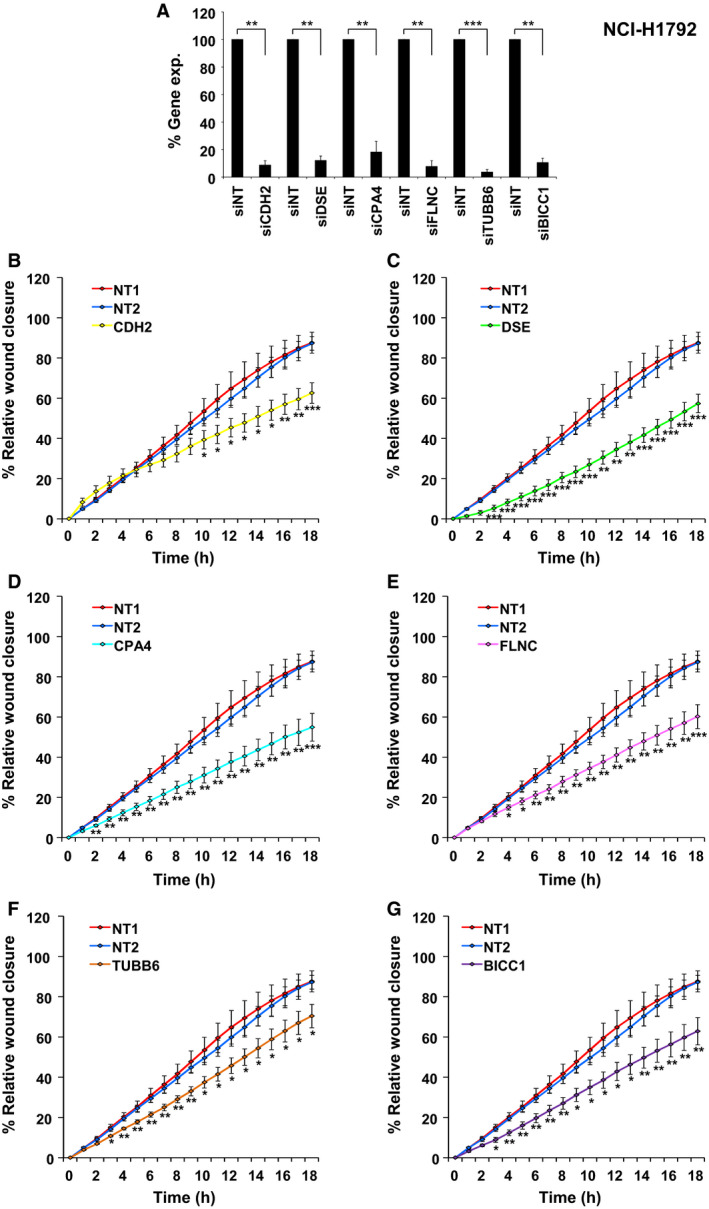
Effect of candidate gene knockdown on NCI‐H1792 cell migration. (A) Knockdown verification by real‐time qPCR (*n* = 3). Expression in cells treated with the nontargeting (NT) siPOOL was normalized to 100%. All genes show effective knockdown by siPOOLs. Statistical test: *t*‐test with Welch’s correction, *P*‐values:   **P < 0.01, ***P < 0.001. (B‐G) Scratch assay results for knockdown of candidate genes (*n* = 4)—(B) *CDH2*, (C) *DSE*, (D) *CPA4*, (E) *FLNC*, (F) *TUBB6*, and (G) *BICC1*. NT1 and NT2 denote technical replicates of siNT (nontargeting siRNA). Mean and SEM are shown. *F*‐test followed by *t*‐test was done for each time point, *P*‐values: *P < 0.05, **P < 0.01, ***P < 0.001. A significant reduction in migration was observed upon knockdown of all six candidate genes.

Knockdown of the six selected genes with specific siPOOLs, complex mixtures of 30 siRNAs to minimize potential off‐target effects [54], led to effective silencing of these genes (Fig. 5A). Time‐resolved IncuCyte^®^ migration assays uncovered a significant impact of each of the knockdown of the selected genes with a significant decrease in the cell migration of NCI‐H1792 cells (Fig. 5B‐G). To further verify that this effect was independent of a specific cellular background, we repeated these experiments in four additional LUAD cell lines (NCI‐H2009, NCI‐H1666, HCC‐827, and NCI‐H838). In all cases, we found effective knockdown of the target genes by siPOOLs (Fig. S4A,5A,6A,7A). For three of the selected hits, we generally observed significantly decreased cell migration upon their knockdown in all five cell lines, for two hits (incl. CDH2) in four cell lines and for one hit in three cell lines (Fig. 5B‐G and S4‐7, Table S9).

To again exclude that cell proliferation might bias the obtained results for migration, two control experiments were performed. First, the proliferation rate of NCI‐H1792 and NCI‐H2009 cells was determined after knockdown of the selected genes. Gene knockdown did not cause any significant difference in proliferation within 48 h—the maximum transfection and migration period of the ORIS™ assays, while the IncuCyte^®^ assays lasted only for 42 h. After 72 h, no differences were observed in NCI‐H2009 cells and only marginally decreased proliferation for BICC1 in NCI‐H1792 (Figs S8A‐B and S9A,B). Second, cell migration assays were performed in the presence of cell cycle inhibitor mitomycin C (MitC) to uncouple effects on cell migration from potential effects on cell proliferation. In the presence of mitomycin C, knockdown of individual genes continued to significantly reduce cell migration in NCI‐H1792 cells (Fig. S8C‐H). In NCI‐H2009 cells, also all six gene knockdowns decreased cell migration reaching statistical significance for *CPA4*, *FLNC,* and *DSE* (Fig. S9C‐H).

Taken together, the effective knockdown of all six candidate genes—including five hits not previously linked to lung cancer cell migration—uncovered their significant impact on lung cancer cell migration in multiple cell lines and independent of cell proliferation.

### 3.5 Filamin family member *FLNC* specifically regulates NSCLC migration

Knockdown of *FLNC* led to reduced migration in all five cell lines without an effect on proliferation. We further investigated FLNC because it showed the highest difference in survival between high‐ versus low‐expressing NSCLC patients (Fig. 4J, HR = 1.6).

**Fig. 6:**
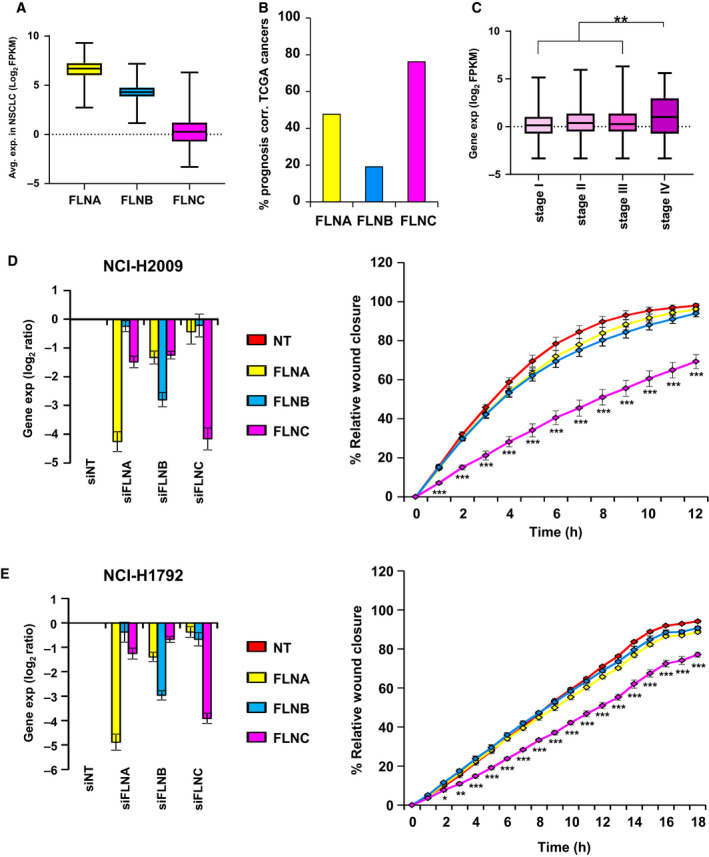
Filamin C (*FLNC*) is an important mediator of NSCLC cell migration. (A) Gene expression (RNA‐seq) of *FLNA* (yellow), *FLNB* (blue), and *FLNC* (pink) in primary human NSCLC samples (*n* = 994, TCGA). (B) Percentage of TCGA cancer types for which *FLNA*, *FLNB,* and *FLNC* expression, respectively, was associated significantly with poor prognosis. (C) Box plot depicting *FLNC* expression in different NSCLC stages (I to IV, *n* = 994, TCGA). Statistical test: one‐way ANOVA followed by Dunnett’s test. *P*‐value for test of trend: **P < 0.01. (D‐E) Knockdown (RT‐qPCR) of *FLNA*, *FLNB,* and *FLNC* and subsequent migration assay in NCI‐H2009 cells (D) and NCI‐H1792 cells (E). All experiments were performed in biological triplicates. Expression in cells treated with the nontargeting (NT) siPOOL was normalized to 100%. Mean and SEM are shown. For RT‐qPCR data, *t*‐test with Welch’s correction was done, *P*‐values: *P < 0.05, **P < 0.01, ***P < 0.001. For scratch assay, *f*‐test and *t*‐test were done for each time point, *P*‐values: *P < 0.05, **P < 0.01, ***P < 0.001. Only knockdown of *FLNC* led to a significant reduction in migration of both cell lines.

*FLNC* is a member of the filamin family comprising the closely related members *FLNA*, *FLNB,* and *FLNC*. In NSCLC patients (TCGA), the overall expression of *FLNC* was found lower compared with the higher expressed *FLNA* and *FLNB* (Fig. 6A). However, a pan‐cancer analysis for the survival association in different tumor entities revealed that *FLNC* expression was associated with poor prognosis in 16 different types of cancers including NSCLC (Figs 6B and 4J). In contrast, the more highly expressed family members *FLNA* and *FLNB* were far less frequently associated with a poor prognosis (Fig. 6B) and had no significant prognostic value in NSCLC patients (Fig. S10A,B). Notably, *FLNC* expression was significantly increased in stage IV of NSCLC compared with earlier stages, so the *FLNC* enrichment to the metastatic stage further linked it to cell migration (Fig. 6C). Again, FLNA and FLNB did not show any stage‐specific patterns (Fig. S10C,D).

To finally assess the impact of the three filamin family members on cell migration, we effectively and specifically knocked them down in two different cell lines (Fig. 6D,E). While *FLNC* knockdown significantly inhibited cell migration, the knockdown of the two other family members had no significant effect on cell migration (Fig. 6D,E).

Altogether, our data show that the least expressed filamin, *FLNC*, has the strongest association with patient survival, is enriched in the metastatic stage IV of NSCLC, and is the only family member to significantly affect lung cancer cell migration.

### 3.6 Differentially expressed circular RNAs in cell migration

The RNA‐seq dataset also enabled the analysis of circular RNAs (circRNAs) based on the analysis of back‐splicing reads [28, 38]. When applying similar filters for minimum expression (detection in >= 50% of the cell lines), regulation (at least threefold), and statistical significance (*P* < 0.05), we identified at the gene level (Table S10) 28 genes generating differentially expressed circRNAs between fast and slow cell lines (Fig. S11A,B, Table S11). At the level of individual circRNAs defined by a specific back‐splice site (Table S12), we found 33 circRNAs to be differentially expressed between fast and slow cell lines (Fig. S11C,D, Table S13). We selected three circRNAs for validation of their regulation by RT‐qPCR using divergent outward‐facing primers and verified the upregulation of circADAMTS6 (exon 2; exon 7) and circAXL (exon 13; exon 14) and the downregulation of circTC2N (exon 4; exon 8) in fast compared with slow cell lines (Fig. S11E,G).

## 4 Discussion

Cell migration is a fundamentally important process in health and disease. Therefore, the systematic evaluation of cell migration and identification of genes controlling this process in a high‐throughput fashion is required, but has so far been a difficult task. In our study, we designed and implemented an effective method called MigExpress to successfully tackle this challenge. MigExpress (identification of Migration control genes by differential Expression) combines two quantitative analyses: cell migration and molecular profiling. The cell migration analysis is performed in parallel for a large number of cell lines using the robust and quantitative ORIS™ assay. The cell lines can also be grown on different matrix surfaces to monitor their migratory behavior based on extracellular signaling and attachment. The molecular profiling is performed by subjecting the same panel of cell lines to transcriptomics (e.g., strand‐specific RNA sequencing after ribosomal RNA depletion) and / or proteomics (e.g., mass spectrometry). Genes differentially expressed between fast and slowly migrating cell lines can then be identified as candidate genes controlling cell migration and can then be individually scrutinized for validation.

Metastasis is the leading cause of death in most cancers, and it occurs when cancer cells migrate from the primary site and form new tumor nodules at a secondary site [55]. Yet, our understanding of the biology of metastasis and the underlying cancer cell migration is far from complete. Lung cancer is one of the most metastatic tumor types and the leading cause of cancer‐related deaths worldwide [7]. Indeed, cell migration is an integral part of NSCLC progression [56, 57, 58]. Therefore, we applied MigExpress on a panel of 54 non‐small cell lung carcinoma (NSCLC) cell lines since NSCLCs represent the most common type of lung cancer [8]. We performed the phenotypic quantification using the ORIS™ assay on three different matrix surfaces to classify the 54 NSCLC cell lines into 18 fast‐, 10 medium‐, and 26 slow‐migrating lines.

The established standard assays for analyzing cell migration are the scratch / wound‐healing assay or the Boyden chamber assay. However, both methods come with limitations—the scratch assay has limited reproducibility due to the difficulties in producing even scratches and the Boyden chamber assay is time‐consuming and cannot be used for high‐throughput studies [59]. We find that assaying migration using ORIS™ in a high‐throughput manner yields robust, reproducible, and quantitative results. Importantly, the results correlate very well with the standard scratch assay.

Regarding the surface coating, we observe that the migration capacities of the NSCLC cell lines are largely independent of the matrix surface, which suggests that cancer cells have inherent molecular properties that render them more migratory, which may not be influenced by ECM signaling. For example, a p53 mutation increases the migration capacity of cells by regulating Rho GTPase family proteins [60, 61].

For the molecular profiling, we evaluated the expression levels of 58 096 transcripts, 148 811 circRNAs, and 9304 proteins using replicates of RNA‐seq and mass spectrometry, respectively, in all 54 cell lines. The observed expression levels between transcriptomics and proteomics correlated very well, while the overlap in candidate hits was lower due to the stringent expression cutoff in both analyses. Thereby, we identified genes differentially regulated between fast‐ and slowly migrating cells. This reflects two important advantages of MigExpress: (a) It covers the entire transcriptome and / or proteome allowing the identification of a broad spectrum of genes at the RNA (both protein‐coding and noncoding) and protein levels, and (b) it employs a large number of cell lines allowing the identification of generally or broadly relevant factors not restricted to single‐cell lines. Indeed, this comprehensiveness is unique among the previously described screens, which were either restricted by a small number of cell lines and / or the limited number of genes queried [15, 16, 17, 18, 19, 20, 21, 22, 23, 24, 25]. Vice versa, the stringent selection criteria applied here should minimize the occurrence of false positives, but increase the likelihood of false negatives such that also important migration factors will be missed by MigExpress.

The statistical analysis identified 84 and 90 differentially regulated candidate molecules between fast‐ and slow‐migrating NSCLC cell lines at the RNA and protein level, respectively. Hence, transcriptomics and proteomics identified comparable numbers of candidates, although the transcriptomic analysis quantified a larger number of genes due to its higher sensitivity but many of these were lowly expressed and hence filtered out in our stringent analysis. GO analysis of the genes upregulated in fast cell lines revealed the significant enrichment of several terms such as ECM organization, signal transduction, cell migration, and cell adhesion [62, 63, 64]. Thus, this analysis validates the MigExpress approach as it significantly enriches genes already implicated to cell migration and thereby reproduces numerous prior studies.

Beyond this state of the art, MigExpress importantly also identifies genes not previously linked to cell migration. To further validate these novel players in lung cancer cell migration, we selected five candidates—DSE, CPA4, FLNC, TUBB6, and BICC1—and the positive control CDH2, which were upregulated in fast cells and also associated with poor overall survival in NSCLC patients. Indeed, all of these genes possess strong pro‐migratory properties. The siPOOL‐mediated knockdown of these genes in five different NSCLC cell lines reduced the cell migration capacities to varying degrees. Notably, this effect was independent of cell proliferation, which was not significantly affected by the knockdown, nor did mitomycin C treatment affect the observed migration phenotypes.

Filamin C (FLNC) is one migration factor from our MigExpress study with a striking impact. To further evaluate the specificity of our findings, we carefully looked at the filamins—a family of actin‐binding proteins that enhance the stability of the cytoskeleton and are involved in mechanotransduction, cell adhesion, and migration [65]. They comprise three closely related members—FLNA, FLNB, and FLNC with ~ 70% homology between their protein sequences [66]. MigExpress identifies FLNC, but not FLNA and FLNB, among the top candidate genes. While FLNA and FLNB are highly expressed in all tissues, FLNC is specifically expressed in lung and muscle tissues (Human Protein Atlas). Multiple studies report a role for FLNA and FLNB in cancer cell migration, but the role of FLNC remains largely unexplored [67, 68, 69, 70, 71, 72]. Here, we observe that FLNC is the least expressed filamin family member in NSCLC, but strikingly it also is the only family member significantly affecting NSCLC cell migration, significantly associating with poor prognosis in NSCLC patients and showing increased expression in metastatic stage IV of NSCLC. In line with our findings, FLNC has been proposed to be associated with poor prognosis in glioblastoma, esophageal squamous cell carcinoma, and prostate cancer [73, 74]. In summary, our study suggests that the MigExpress candidate FLNC, rather than FLNA or FLNB, is a relevant factor in NSCLC migration, metastasis, and survival.

As a future outlook, similar approaches combining the molecular profiling and functional screening of larger sets of cell lines can also be used to discover new factors governing other cellular properties such as proliferation, apoptosis, or drug response or the impact of molecular aberrations such as canonical or noncanonical mutations on the expression of cancer genes [75, 76, 77].

## 5 Conclusions

In summary, we developed, applied, and validated MigExpress as a method to systematically identify genes impacting the highly relevant process of cell migration by combining quantitative molecular profiling with robust quantification of cell migration in a broad panel of cell lines. As a proof of principle, we successfully discovered and validated multiple genes essential in lung cancer cell migration. Future studies will need to unravel whether these factors differentially expressed and regulating migration in cell culture correlate with the metastatic potential *in vivo*. As a comprehensive resource, we provide a deep transcriptomic and proteomic profile of a NSCLC cell line panel and a quantitative assessment of their proliferation and migration properties. For the future, MigExpress can be broadly employed to uncover migration control genes across all fields of life science research.

## Conflict of interest

S.Di. is co‐owner of siTOOLs Biotech GmbH, Martinsried, Germany. All other authors disclose no competing interests.

## Author contributions

JP analyzed, validated, and extended the migration screen; ACB performed and analyzed the migration screen; SDh, JS, and MA conducted and analyzed experiments; YS analyzed the transcriptome data; JB, CM, CL, and BK performed and analyzed the proteome data; SDi conceived, coordinated, and supervised the project; and JP and SDi wrote the manuscript and all authors read, edited, and approved the manuscript.

## Ethics approval and consent to participate

Not applicable.

## Consent for publication

Not applicable.

## Supporting information


**Fig. S1**. Migration assay validation. A. Standard error of mean (SEM) plotted for replicate experiments of each cell line grown on three different coated matrices. The mean is represented by red solid line. The red dashed line represents SEM of 5. B. Lack of correlation between proliferation of the 54 NSCLC cell lines at 72 h and the percentage migration from ORIS™ assay (uncoated). R represents Pearson’s correlation coefficient. C. Representative images of 18 further fast and slow cell lines used for validation in the IncuCyte^®^ scratch assay at 0 h, 24 h and 48 h.
**Fig. S2**. Gene expression validation in fast versus slow NSCLC cell lines. A‐C. Genes downregulated in fast cell lines compared to slow cell lines from RNA‐seq (*n* = 54) and RT‐qPCR (*n* = 16) data – A. *CEACAM6*, B. *PRR15L*, C. *AGR2*. D‐F. Genes previously reported as regulators of NSCLC cell migration – D. *VIM*, E. *LOXL2*, F. *CDH1*. Statistical test: *t*‐test with Welch’s correction, *P*‐values: *P < 0.05, **P < 0.01, ***P < 0.001.
**Fig. S3**. Gene Ontology (GO) analysis for pathway enrichment of candidate genes. A. GO analysis of upregulated genes from RNA‐seq and mass spectrometry data (*n* = 98). B. GO analysis of downregulated genes from RNA‐seq and mass spectrometry data (*n* = 76). All pathways selected are statistically significant (*P*‐value < 0.05) and have a minimum of four genes enriched in the pathway.
**Fig. S4**. Effect of knockdown of candidate genes on NCI‐H2009 cell migration. A. Knockdown verification by real‐time qPCR (*n* = 3). Expression in cells treated with the nontargeting (NT) siPOOL was normalized to 100%. All genes show efficient knockdown. –Statistical test: *t*‐test with Welch’s correction, *P*‐values: *P < 0.05, **P < 0.01, ***P < 0.001. B‐G. Scratch assay results for knockdown of candidate genes (*n* = 4) ‐ B. *CDH2*, C. *DSE*, D. *CPA4*, E. *FLNC*, F. *TUBB6*, G. *BICC1*. NT1 and NT2 denote technical replicates of siNT (nontargeting siRNA). Mean and SEM are shown. *F*‐test followed by *t*‐test was done for each time‐point, *P*‐values: *P < 0.05, **P < 0.01, ***P < 0.001. A significant reduction in migration capacity was observed upon knockdown of the candidate genes except *TUBB6*.
**Fig. S5**. Effect of knockdown of candidate genes on NCI‐H1666 cell migration. A. Knockdown verification by real‐time qPCR (*n* = 3). Expression in cells treated with the nontargeting (NT) siPOOL was normalized to 100%. All genes show efficient knockdown. Statistical test: *t*‐test with Welch’s correction, *P*‐values: *P < 0.05, **P < 0.01, ***P < 0.001. *CDH2* shows efficient knockdown but is not statistically significant. B‐G. Scratch assay results for knockdown of candidate genes (*n* = 4) ‐ B. *CDH2*, C. *DSE*, D. *CPA4*, E. *FLNC*, F. *TUBB6*, G. *BICC1*. NT1 and NT2 denote technical replicates of siNT (non‐targeting siRNA). Mean and SEM are shown. *F*‐test followed by *t*‐test was done for each time‐point, *P*‐values: *P < 0.05, **P < 0.01, ***P < 0.001. A significant reduction in migration capacity was observed upon knockdown of all six candidate genes.
**Fig. S6**. Effect of knockdown of candidate genes on HCC‐827 cell migration. A. Knockdown verification by real‐time qPCR (*n* = 3). Expression in cells treated with the non‐targeting (NT) siPOOL was normalized to 100%. All genes show efficient knockdown. Statistical test: *t*‐test with Welch’s correction, *P*‐values: *P < 0.05, **P < 0.01, ***P < 0.001. *CDH2* knockdown was not statistically significant. B‐G. Scratch assay results for knockdown of candidate genes (*n* = 5) ‐ B. *CDH2*, C. *DSE*, D. *CPA4*, E. *FLNC*, F. *TUBB6*, G. *BICC1*. NT1 and NT2 denote technical replicates of siNT (non‐targeting siRNA). Mean and SEM are shown. *F*‐test followed by *t*‐test was done for each time‐point, *P*‐values: *P < 0.05, **P < 0.01, ***P < 0.001. A significant reduction in migration capacity was observed upon knockdown of the candidate genes ‐ *DSE*, *FLNC* and *BICC1*.
**Fig. S7**. Effect of knockdown of candidate genes on NCI‐H838 cell migration. A. Knockdown verification by real‐time qPCR (*n* = 3). Expression in cells treated with the non‐targeting (NT) siPOOL was normalized to 100%. All genes show efficient knockdown. Statistical test: *t*‐test with Welch’s correction, *P*‐values: *P < 0.05, **P < 0.01, ***P < 0.001. B‐G. Scratch assay results for knockdown of candidate genes (*n* = 3) ‐ B. *CDH2*, C. *DSE*, D. *CPA4*, E. *FLNC*, F. *TUBB6*, G. *BICC1*. NT1 and NT2 denote technical replicates of siNT (non‐targeting siRNA). Mean and SEM are shown. *F*‐test followed by *t*‐test was done for each time‐point, *P*‐values: *P < 0.05, **P < 0.01, ***P < 0.001. A significant reduction in migration capacity was observed upon knockdown of the candidate genes except *CDH2* and *CPA4*.
**Fig. S8**. Effect of knockdown of candidate genes on NCI‐H1792 cell proliferation and migration. A‐B. Cell proliferation assessed with trypan blue counting at 0 h, 48 h and 72 h after siPOOL‐mediated knockdown of the six candidate genes (*n* = 3). *F*‐test followed by *t*‐test was done for each time‐point, *P*‐values: *P < 0.05, **P < 0.01, ***P < 0.001. Knockdown of all genes show no effect on proliferation for all time points, except for a minimal decrease for siBICC1 condition at 72 h. C‐H. IncuCyte^®^ cell migration assay with siPOOL‐mediated knockdown of candidate genes in the presence of Mitomycin C (7.5–10 µm) for ‐ C. *CDH2*, D. DSE, E. *CPA4*, F. *FLNC*, G. *TUBB6* and H. *BICC1*. NT1 and NT2 denote technical replicates of siNT (non‐targeting siRNA). Mean and SEM are shown. *F*‐test followed by *t*‐test was done for each time‐point, *P*‐values: *P < 0.05, **P < 0.01, ***P < 0.001. A significant reduction in migration capacity was observed upon knockdown of all six candidate genes.
**Fig. S9**. Effect of knockdown of candidate genes on NCI‐H2009 cell proliferation and migration. A‐B. Cell proliferation assessed with trypan blue counting at 0 h, 48 h and 72 h after siPOOL‐mediated knockdown of the six candidate genes (*n* = 3). F‐test followed by *t*‐test was done for each time‐point, *P*‐values: *P < 0.05, **P < 0.01, ***P < 0.001. Knockdown of all genes show no effect on proliferation for all time points. C‐H. IncuCyte^®^ cell migration assay with siPOOL‐mediated knockdown of candidate genes in the presence of Mitomycin C (7.5–10 µm) for ‐ C. *CDH2*, D. DSE, E. *CPA4*, F. *FLNC*, G. *TUBB6* and H. *BICC1*. NT1 and NT2 denote technical replicates of siNT (non‐targeting siRNA). Mean and SEM are shown. F‐test followed by *t*‐test was done for each time‐point, *P*‐values: *P < 0.05, **P < 0.01, ***P < 0.001. A significant reduction in migration capacity was observed upon knockdown of *DSE*, *CPA4* or *FLNC*, while a non‐significant trend to reduction was noted for the other candidates.
**Fig. S10**. Survival and staging analyses of *FLNA* and *FLNB* expression in patient samples. A. Kaplan‐Meier survival plot of TCGA NSCLC patient samples with *FLNA* high versus low expression. Log rank‐test *P*‐value is not significant. B. Kaplan‐Meier survival plot of TCGA NSCLC patient samples with *FLNB* high versus low expression. Log rank‐test *P*‐value is not significant. C. Expression of *FLNA* in different stages of NSCLC patient samples from TCGA. D. Expression of *FLNB* in different stages of NSCLC patient samples from TCGA. Statistical test: One‐way ANOVA followed by Dunnett’s test not significant. *P*‐value for test‐of‐trend is not significant.
**Fig. S11**. circRNA profiling of NSCLC cell lines. A. Pipeline for the identification of genes with differential circRNA expression (gene level analysis) from RNA‐seq data. The analysis identified 28 candidate circRNAs based on expression, regulation and significance at the gene level. B. Volcano plot for 4072 genes that have a minimum expression of circRNAs detected in ≥ 50% of cell lines. The red dots represent genes with significantly upregulated circRNAs (fold change ≥ 3, *P* < 0.05) and the green dots represent genes with significantly downregulated circRNAs (fold change ≤ 0.333, *P* < 0.05). Candidates selected for validation are indicated with larger circles. C. Pipeline for the identification of differentially expressed circRNAs (back‐splice level analysis) from RNA‐seq data. The analysis identified 33 individual differentially expressed circRNAs based on expression, regulation and significance. D. Volcano plot for 5050 circRNAs that have been detected in ≥ 50% of cell lines. The red dots represent significantly upregulated circRNAs (fold change ≥ 3, *P* < 0.05) and the green dots represent significantly downregulated circRNAs (fold change ≤ 0.333, *P* < 0.05). Candidates selected for validation are indicated with larger circles. E‐G. RT‐qPCR validation of selected circRNA candidates at the back‐splice level in eight fast versus eight slow cell lines. The RNA‐seq level expression is also provided for the same cell lines. Statistical test: *t*‐test with Welch’s correction, *P*‐values: *P < 0.05, **P < 0.01, ***P < 0.001.

**Table S1**. siPOOLs. Sense and antisense sequences of 30 siRNAs used against each gene ‐ *CDH2*, *DSE*, *CPA4*, *FLNC*, *TUBB6*, *BICC1*, *FLNA* and *FLNB*.
**Table S2**. Primers. Forward and reverse primer sequences for all genes tested by real time‐qPCR.
**Table S3**. Summary of ORIS™ assay for 54 NSCLC cell lines. Values for the assay were calculated for each cell line grown on each matrix surfaces. The table contains average values from all biological replicates. Orange, blue and green represents slow, medium and fast cell lines.
**Table S4**. RNA‐seq data of 54 NSCLC cell lines. RNA‐seq (log_2_FPKM) expression data for 58096 genes for 54 NSCLC cell lines. Fold change values, significance and correlations with different coatings and proliferation for each gene are also provided.
**Table S5**. Candidate genes from RNA‐seq data regulated between fast and slow cell lines. List of 84 candidate genes obtained from RNA‐seq data through the filtration criteria described (Figure 3A). Log_2_FPKM values provided for 18 fast and 26 slow cell lines.
**Table S6**. RT‐qPCR validation of RNA‐seq data. Total of 41 genes were tested in 8 fast and 8 slow cell lines by RT‐qPCR. F‐test followed by *t*‐test was done for fast versus slow cell lines. All genes are significantly differentially regulated in the RNA‐seq data.
**Table S7**. Mass spectrometry data of 54 NSCLC cell lines. Protein expression data for 9304 genes for 54 NSCLC cell lines. Fold change values, significance and correlations with different coatings and proliferation for each gene are also provided.
**Table S8**. Candidate genes from mass spectrometry data regulated between fast and slow cell lines. List of 90 candidate genes obtained from mass spectrometry data through the filtration criteria described (Figure 3E). Expression values provided for 18 fast and 26 slow cell lines.
**Table S9**. Summary of analyses with candidate genes – *CDH2*, *DSE*, *CPA4*, *FLNC*, *TUBB6* and *BICC1*.
**Table S10**. circRNA expression analysis at the gene level from RNA‐seq data of 54 NSCLC cell lines. Back splicing reads per gene normalized to the sequencing library size for 12251 genes in 54 NSCLC cell lines [28, 38]. Fold change values and significance comparing fast versus slowly migrating cell lines on different coating conditions for each gene are also provided.
**Table S11**. Differentially expressed circRNAs at the gene level regulated between fast and slow cell lines. List of 28 genes obtained from RNA‐seq data with differential circRNA expression selected through the filtration criteria described (Supplementary figure 11A). Expression values are provided for 18 fast and 26 slow cell lines which showed these fast or slow properties on the majority of coatings.
**Table S12**. circRNA expression analysis at the back‐splice level from RNA‐seq data of 54 NSCLC cell lines. Back splicing reads per individual circRNA (back‐splice site) normalized to the sequencing library size for 148811 circRNAs in 54 NSCLC cell lines [28, 38]. Fold change values and significance comparing fast versus slowly migrating cell lines on different coating conditions for each circRNA are also provided.
**Table S13**. Differentially expressed circRNAs regulated between fast and slow cell lines detected at the back‐splice level. List of 33 candidate circRNAs obtained from RNA‐seq data and selected through the filtration criteria described (Figure S11C). Expression values provided for 18 fast and 26 slow cell lines which showed these fast or slow properties on the majority of coatings.

## Data Availability

The transcriptomic raw data are provided via the GEO database by the dataset identifier GSE160683. The proteomic raw data, MaxQuant search results, and used protein sequence database have been deposited at the ProteomeXchange Consortium via the PRIDE partner repository and can be accessed using the dataset identifier PXD022146.
