# Supplementary material for: Systematic analysis of migration factors by MigExpress identifies essential cell migration control genes in non‐small cell lung cancer
Source: Mol Oncol. 2021 May 14;15(7):1797–817. doi: 10.1002/1878-0261.12973 (PMC8253088; doi:10.1002/1878-0261.12973)
Supplement: Supplementary file 1 — Fig. S1. Migration assay validation. A. Standard error of mean (SEM) plotted for replicate experiments of each cell line grown on three different coated matrices. The mean is represented by red solid line. The red dashed line represents SEM of 5. B. Lack of correlation between proliferation of the 54 NSCLC cell lines at 72 h and the percentage migration from ORIS™ assay (uncoated). R represents Pearson’s correlation coefficient. C. Representative images of 18 further fast and slow cell lines used for validation in the IncuCyte® scratch assay at 0 h, 24 h and 48 h. Fig. S2. Gene expression validation in fast versus slow NSCLC cell lines. A‐C. Genes downregulated in fast cell lines compared to slow cell lines from RNA‐seq (n = 54) and RT‐qPCR (n = 16) data – A. CEACAM6, B. PRR15L, C. AGR2. D‐F. Genes previously reported as regulators of NSCLC cell migration – D. VIM, E. LOXL2, F. CDH1. Statistical test: t‐test with Welch’s correction, P‐values: *P < 0.05, **P < 0.01, ***P < 0.001. Fig. S3. Gene Ontology (GO) analysis for pathway enrichment of candidate genes. A. GO analysis of upregulated genes from RNA‐seq and mass spectrometry data (n = 98). B. GO analysis of downregulated genes from RNA‐seq and mass spectrometry data (n = 76). All pathways selected are statistically significant (P‐value < 0.05) and have a minimum of four genes enriched in the pathway. Fig. S4. Effect of knockdown of candidate genes on NCI‐H2009 cell migration. A. Knockdown verification by real‐time qPCR (n = 3). Expression in cells treated with the nontargeting (NT) siPOOL was normalized to 100%. All genes show efficient knockdown. –Statistical test: t‐test with Welch’s correction, P‐values: *P < 0.05, **P < 0.01, ***P < 0.001. B‐G. Scratch assay results for knockdown of candidate genes (n = 4) ‐ B. CDH2, C. DSE, D. CPA4, E. FLNC, F. TUBB6, G. BICC1. NT1 and NT2 denote technical replicates of siNT (nontargeting siRNA). Mean and SEM are shown. F‐test followed by t‐test was done for each time‐point, [file MOL2-15-1797-s001.pdf]

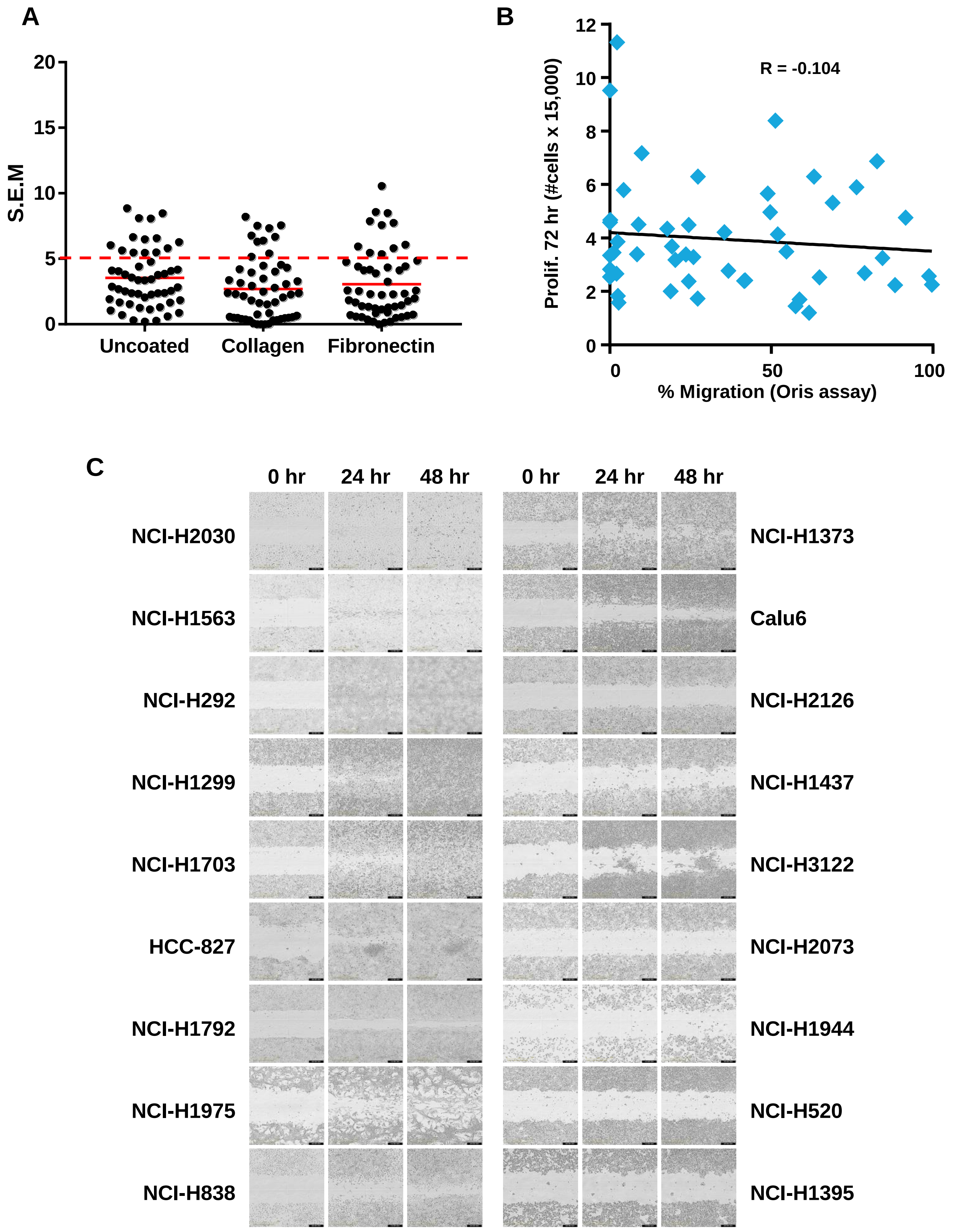

Supplementary figure 1

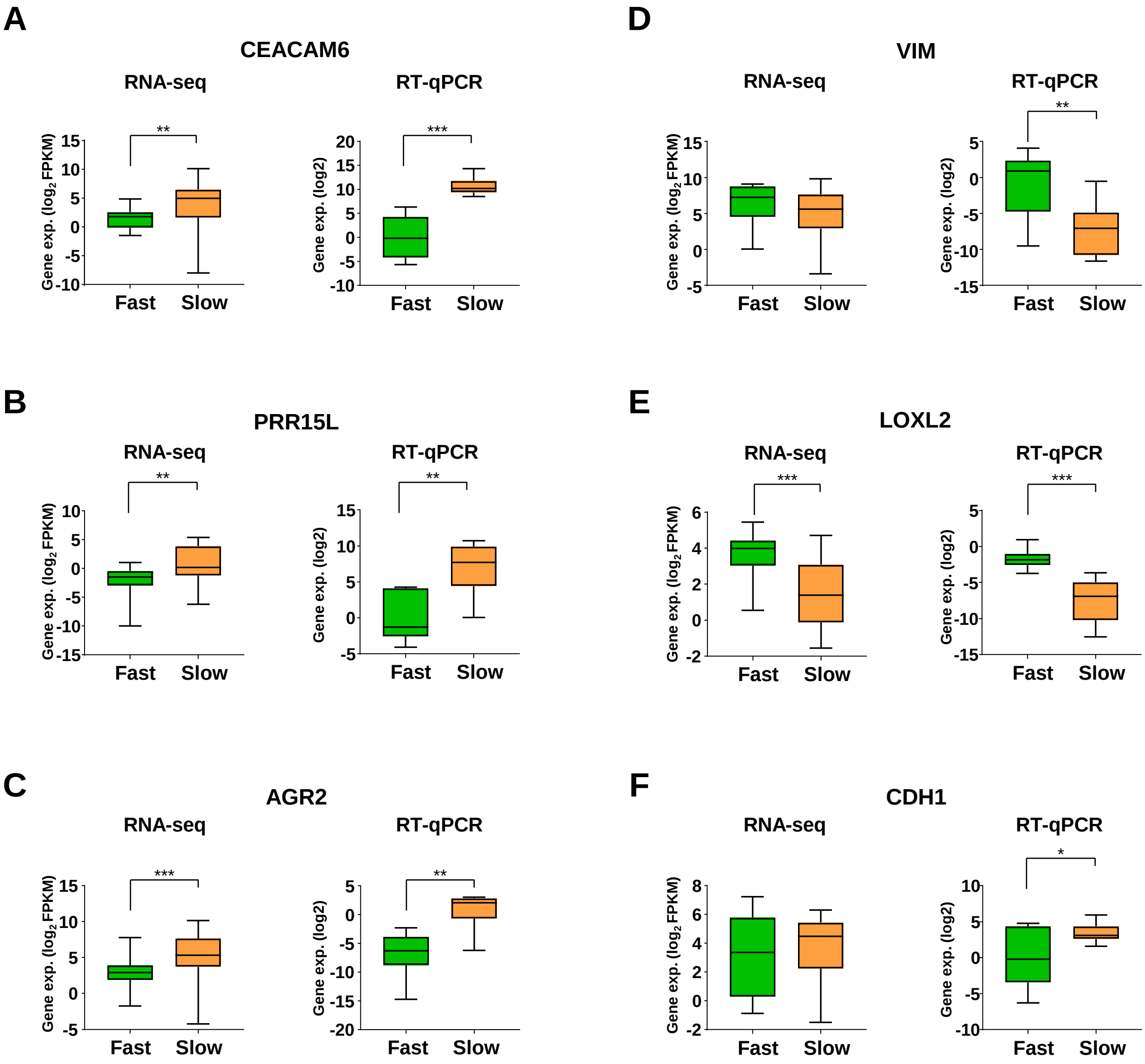

Supplementary figure 2

Genes upregulated in fast cells (98 genes)

A

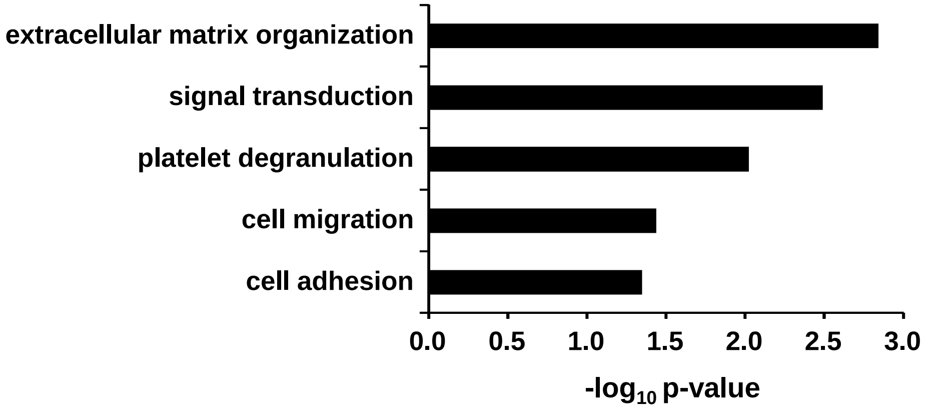

Genes downregulated in fast cells (76 genes)

B

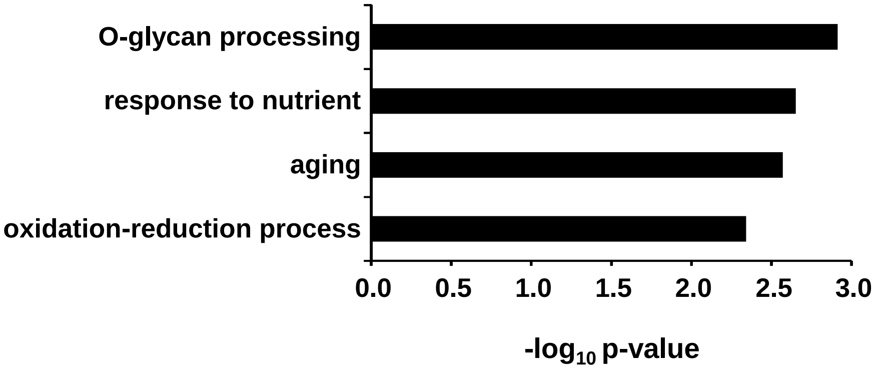

Supplementary figure 3

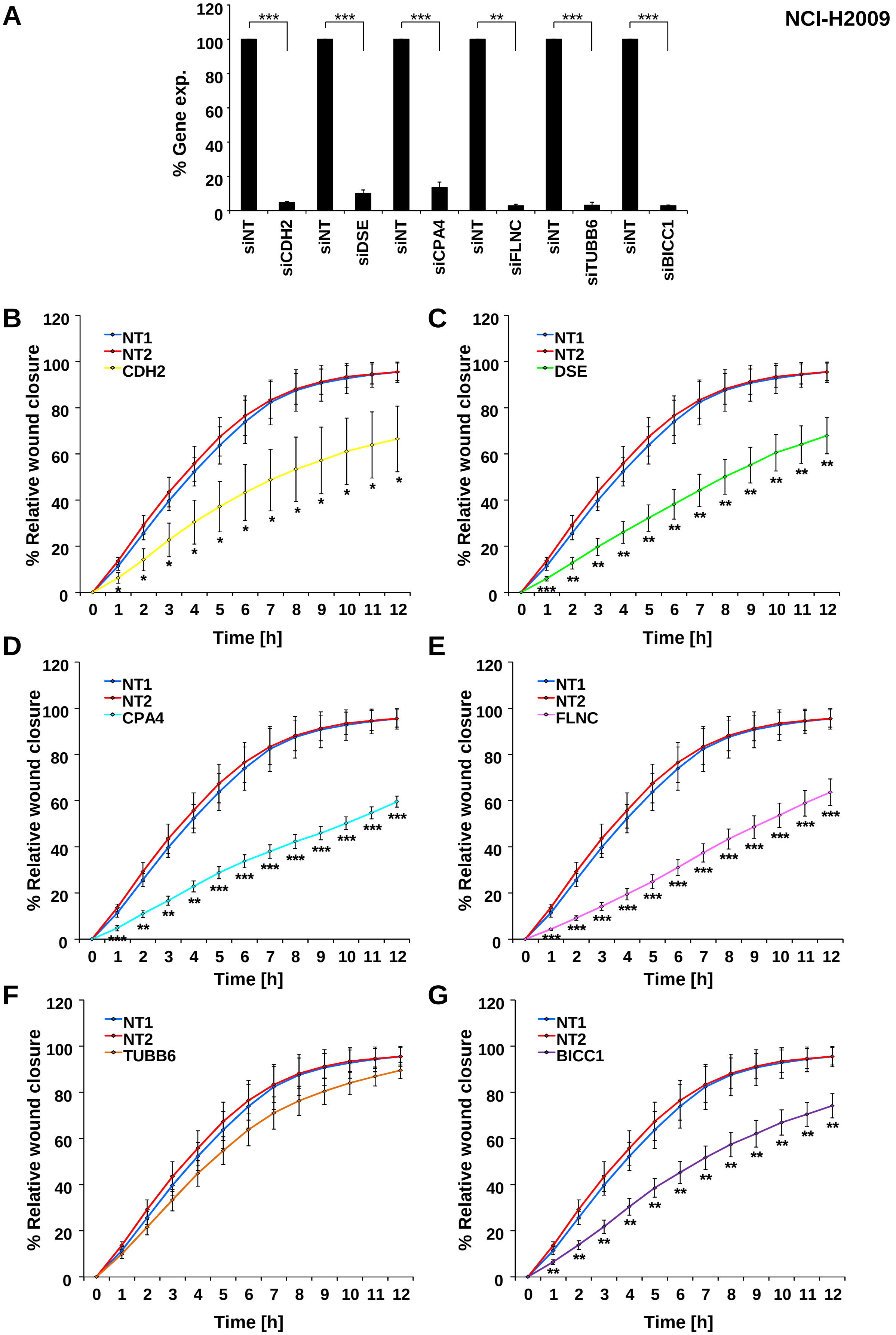

Supplementary figure 4

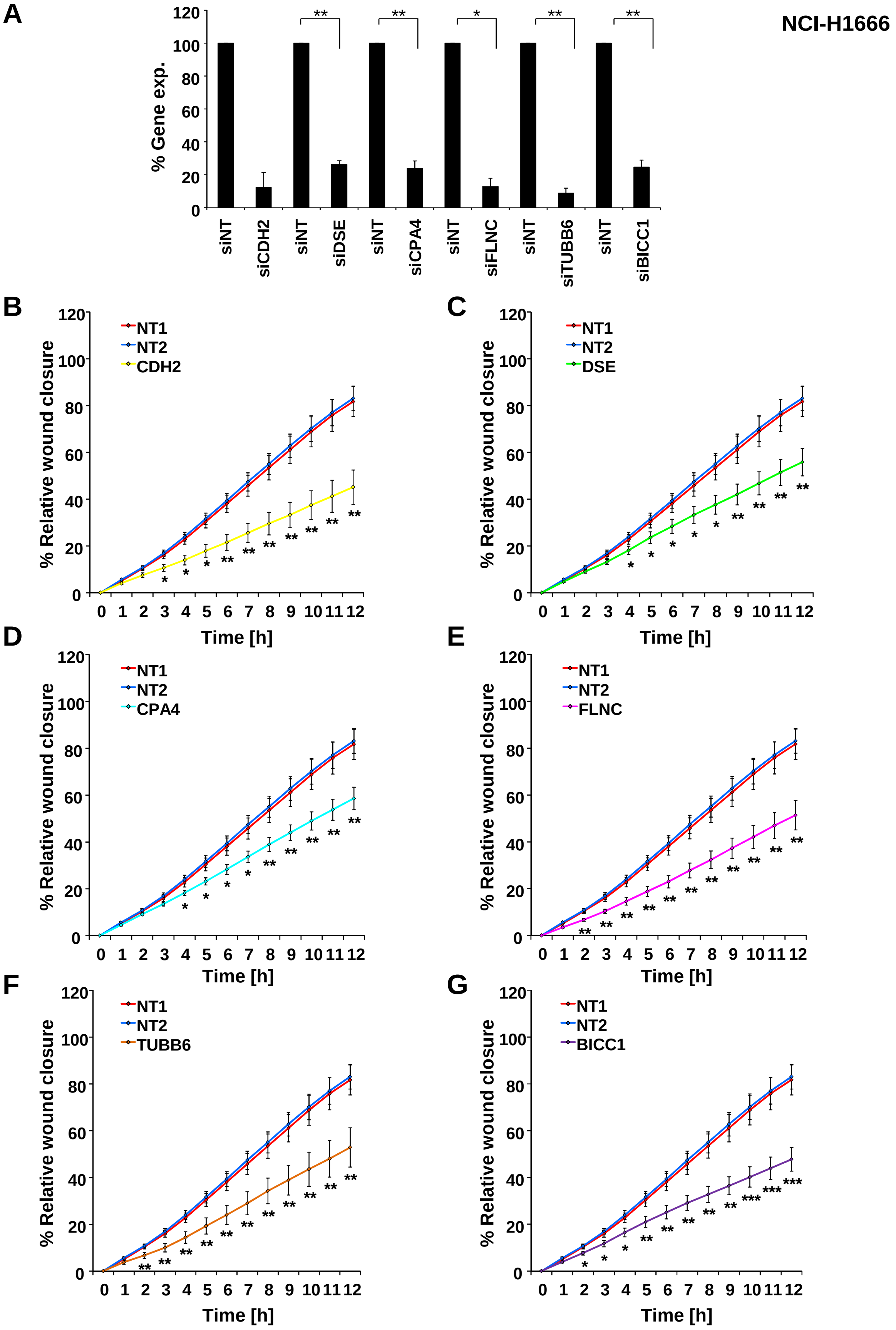

Supplementary figure 5

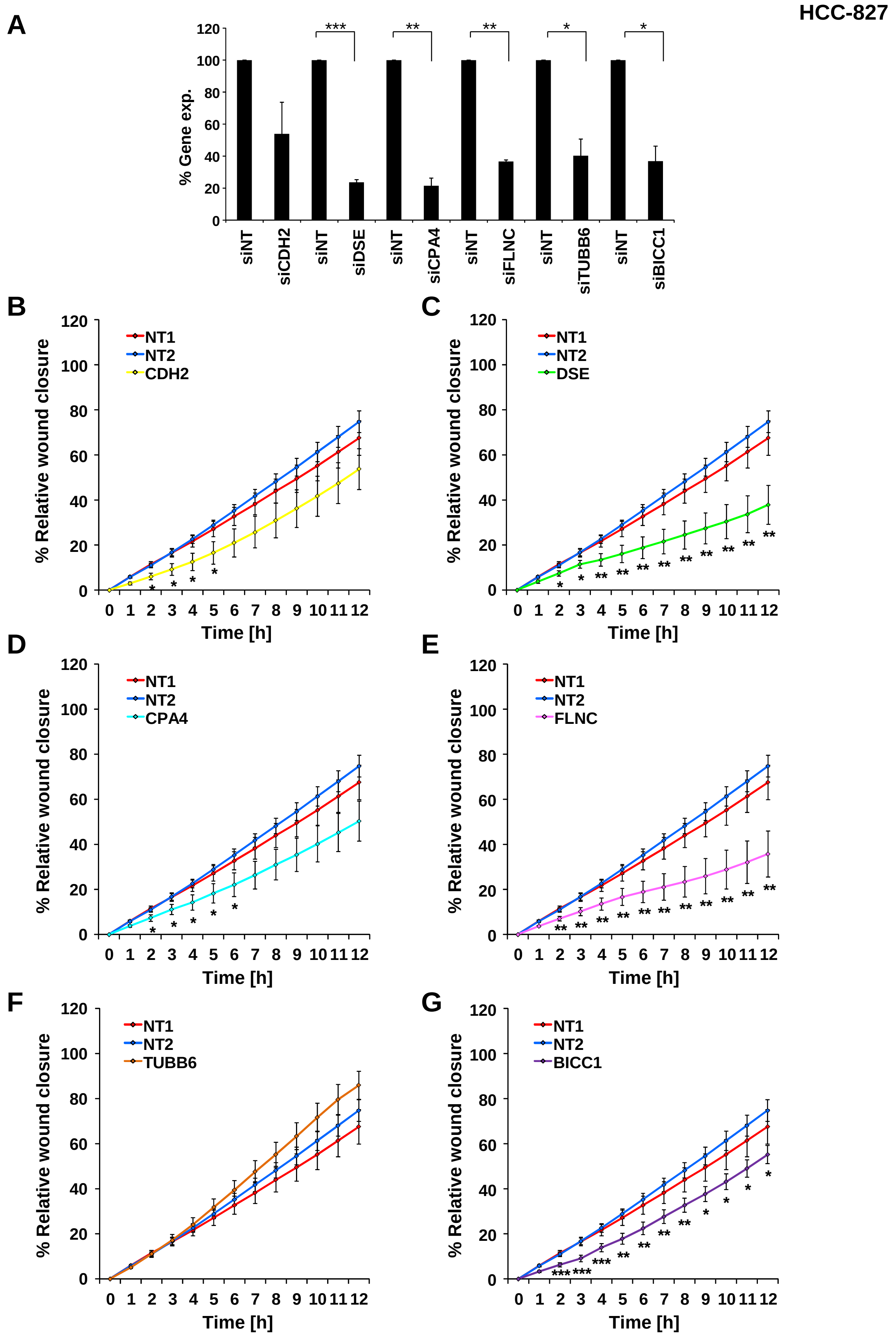

Supplementary figure 6

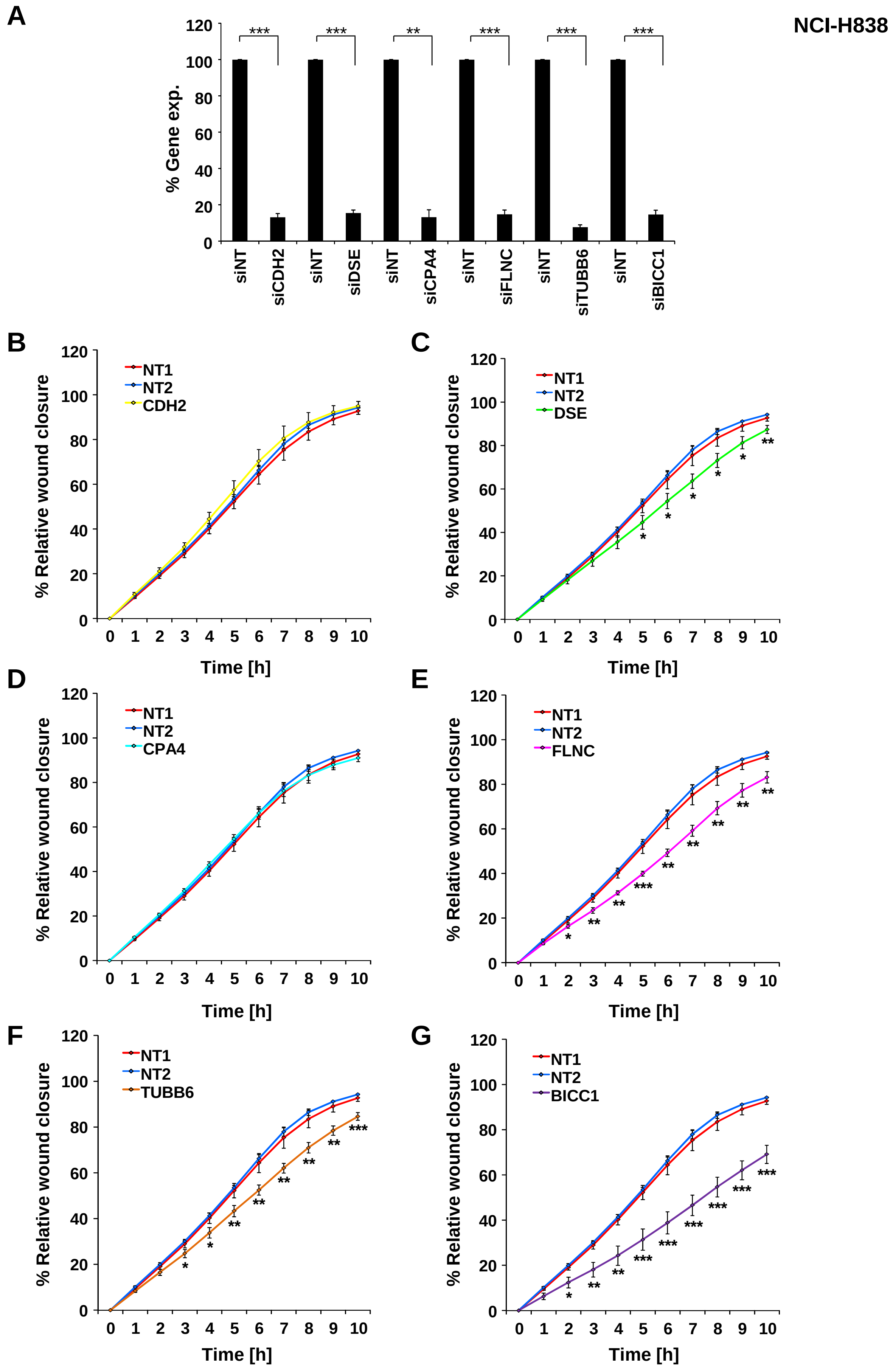

Supplementary figure 7

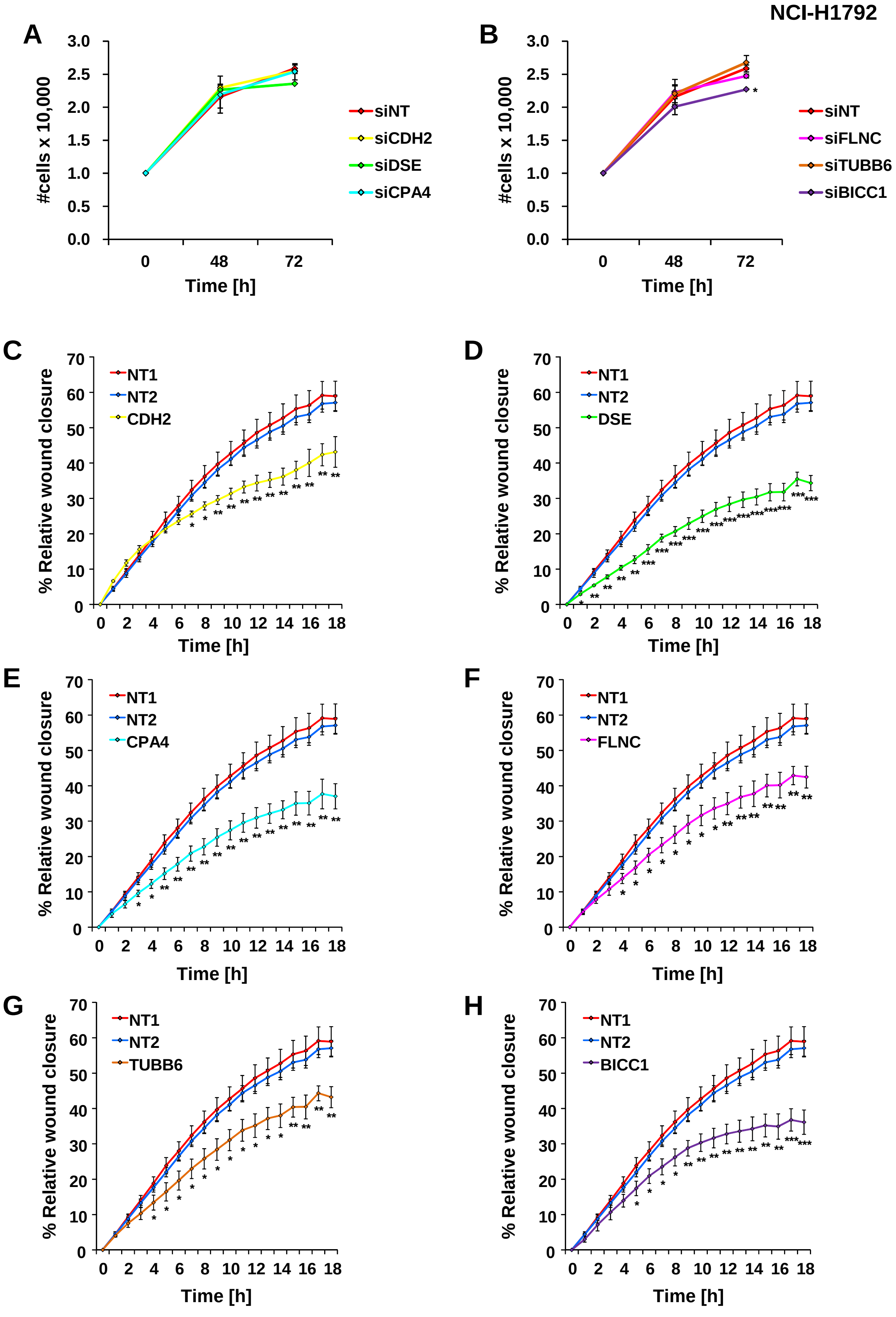

Supplementary figure 8

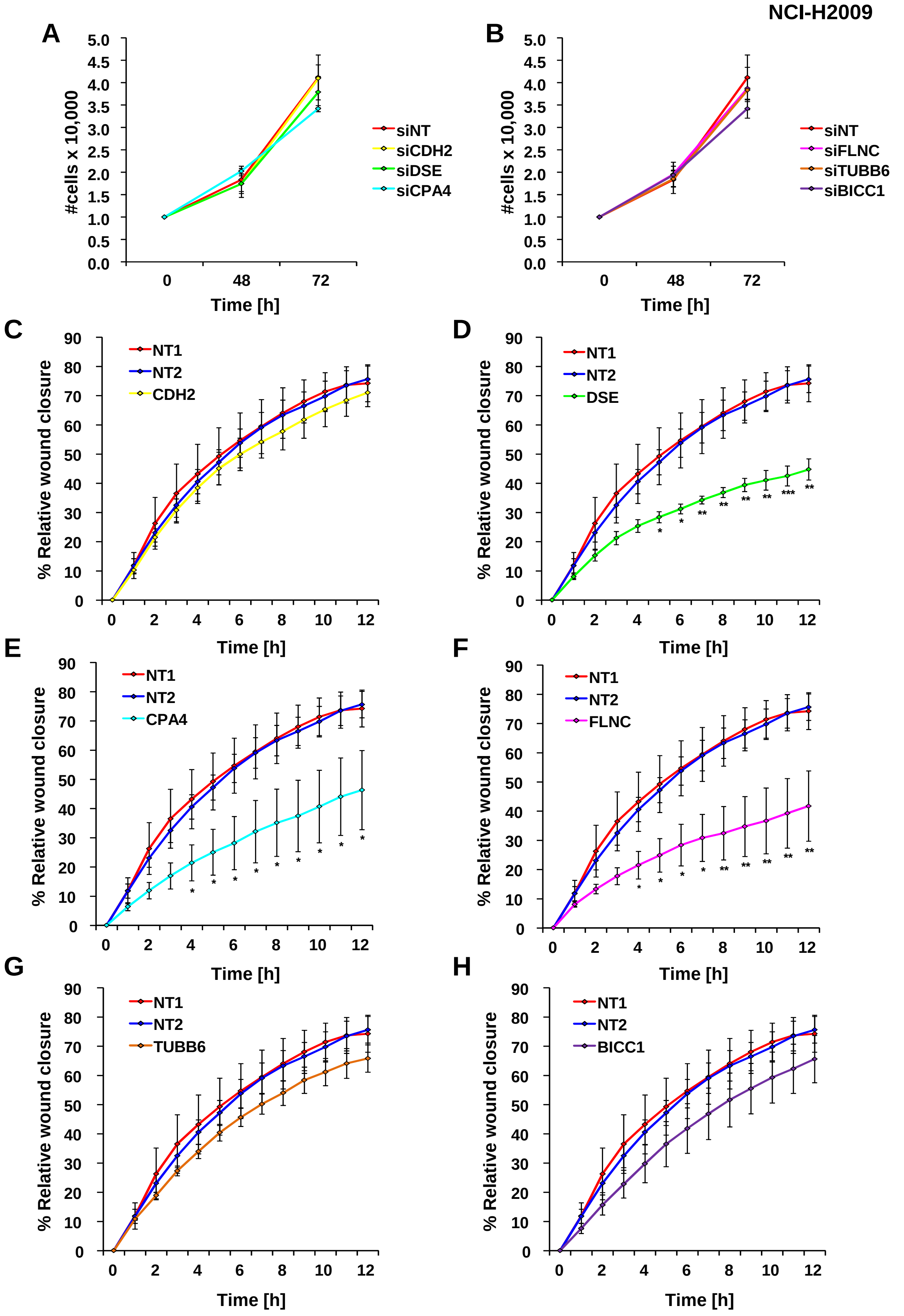

Supplementary figure 9

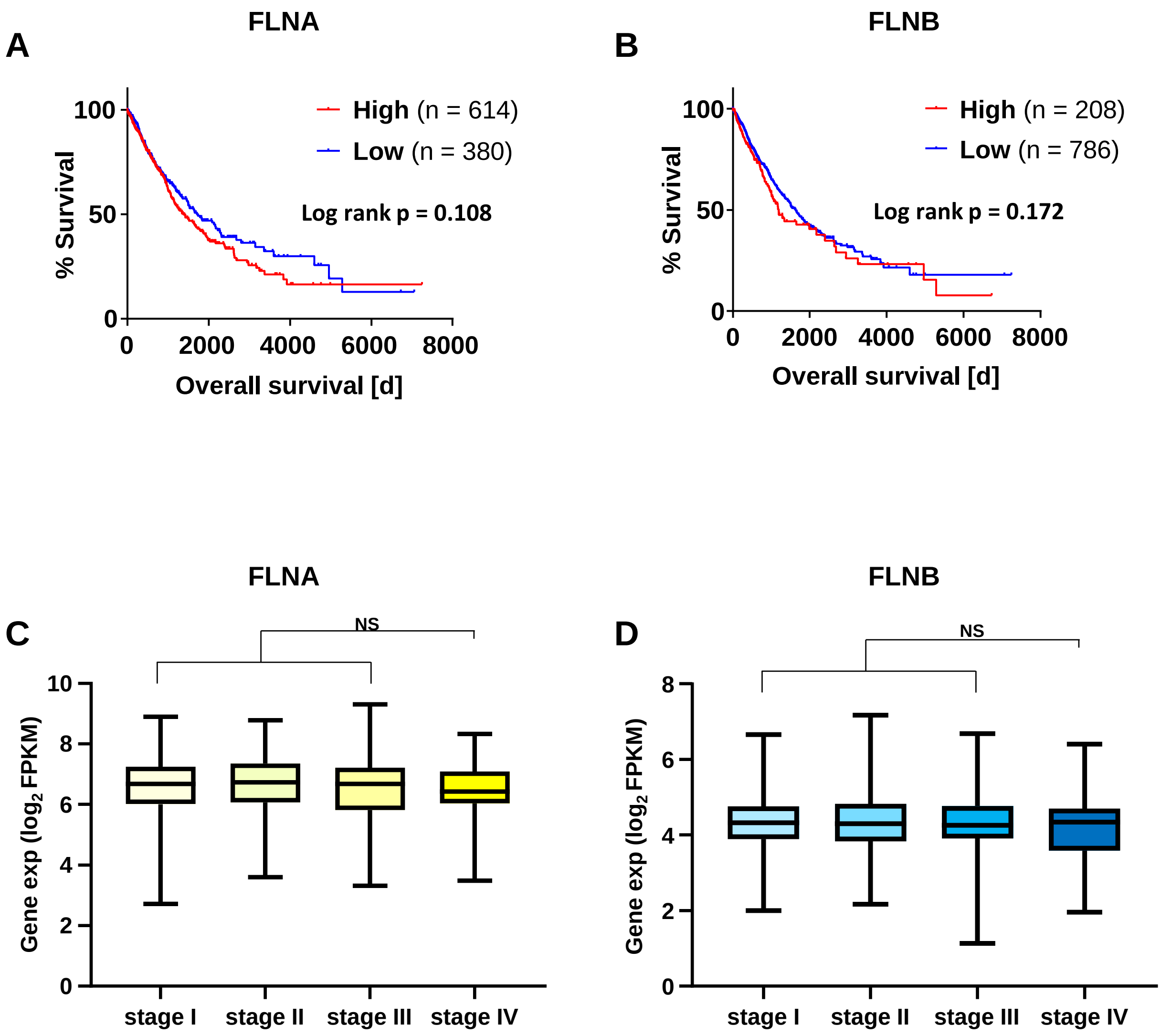

Supplementary figure 10

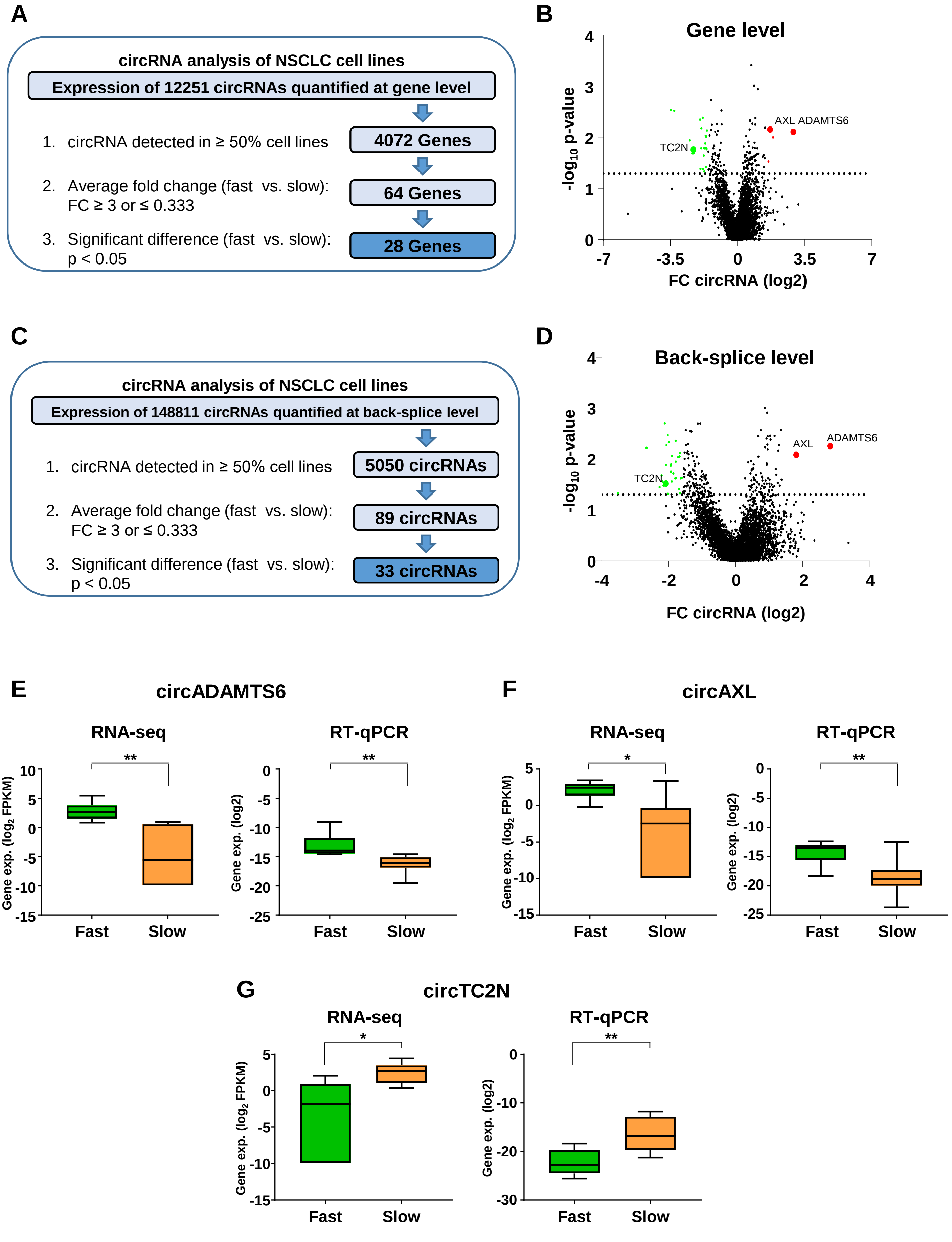

Supplementary figure 11
