# Supplementary material for: Systematic analysis of migration factors by MigExpress identifies essential cell migration control genes in non‐small cell lung cancer
Source: Mol Oncol. 2021 May 14;15(7):1797–817. doi: 10.1002/1878-0261.12973 (PMC8253088; doi:10.1002/1878-0261.12973)
Supplement: Supplementary file 2 — Table S1. siPOOLs. Sense and antisense sequences of 30 siRNAs used against each gene ‐ CDH2, DSE, CPA4, FLNC, TUBB6, BICC1, FLNA and FLNB. Table S2. Primers. Forward and reverse primer sequences for all genes tested by real time‐qPCR. Table S3. Summary of ORIS™ assay for 54 NSCLC cell lines. Values for the assay were calculated for each cell line grown on each matrix surfaces. The table contains average values from all biological replicates. Orange, blue and green represents slow, medium and fast cell lines. Table S4. RNA‐seq data of 54 NSCLC cell lines. RNA‐seq (log2FPKM) expression data for 58096 genes for 54 NSCLC cell lines. Fold change values, significance and correlations with different coatings and proliferation for each gene are also provided. Table S5. Candidate genes from RNA‐seq data regulated between fast and slow cell lines. List of 84 candidate genes obtained from RNA‐seq data through the filtration criteria described (Figure 3A). Log2FPKM values provided for 18 fast and 26 slow cell lines. Table S6. RT‐qPCR validation of RNA‐seq data. Total of 41 genes were tested in 8 fast and 8 slow cell lines by RT‐qPCR. F‐test followed by t‐test was done for fast versus slow cell lines. All genes are significantly differentially regulated in the RNA‐seq data. Table S7. Mass spectrometry data of 54 NSCLC cell lines. Protein expression data for 9304 genes for 54 NSCLC cell lines. Fold change values, significance and correlations with different coatings and proliferation for each gene are also provided. Table S8. Candidate genes from mass spectrometry data regulated between fast and slow cell lines. List of 90 candidate genes obtained from mass spectrometry data through the filtration criteria described (Figure 3E). Expression values provided for 18 fast and 26 slow cell lines. Table S9. Summary of analyses with candidate genes – CDH2, DSE, CPA4, FLNC, TUBB6 and BICC1. Table S10. circRNA expression analysis at the gene level from RNA‐seq data of 54 NSCLC cell lines. [file MOL2-15-1797-s002.docx]

**Table S1**. siPOOLs. Sense and antisense sequences of 30 siRNAs used against each gene - *CDH2*, *DSE*, *CPA4*, *FLNC*, *TUBB6*, *BICC1*, *FLNA* and *FLNB*

**Table S2**. Primers. Forward and reverse primer sequences for all genes tested by real time-qPCR.

**Table S3**. Summary of ORIS™ assay for 54 NSCLC cell lines. Values for the assay were calculated for each cell line grown on each matrix surfaces. The table contains average values from all biological replicates. Orange, blue and green represents slow, medium and fast cell lines.

**Table S4**. RNA-seq data of 54 NSCLC cell lines. RNA-seq (log_2_FPKM) expression data for 58096 genes for 54 NSCLC cell lines. Fold change values, significance and correlations with different coatings and proliferation for each gene are also provided.

**Table S5**. Candidate genes from RNA-seq data regulated between fast and slow cell lines. List of 84 candidate genes obtained from RNA-seq data through the filtration criteria described (Figure 3A). Log_2_FPKM values provided for 18 fast and 26 slow cell lines.

**Table S6**. RT-qPCR validation of RNA-seq data. Total of 41 genes were tested in 8 fast and 8 slow cell lines by RT-qPCR. F-test followed by t-test was done for fast versus slow cell lines. All genes are significantly differentially regulated in the RNA-seq data.

**Table S7**. Mass spectrometry data of 54 NSCLC cell lines. Protein expression data for 9304 genes for 54 NSCLC cell lines. Fold change values, significance and correlations with different coatings and proliferation for each gene are also provided.

**Table S8**. Candidate genes from mass spectrometry data regulated between fast and slow cell lines. List of 90 candidate genes obtained from mass spectrometry data through the filtration criteria described (Figure 3E). Expression values provided for 18 fast and 26 slow cell lines.

**Table S9**. Summary of analyses with candidate genes – *CDH2*, *DSE*, *CPA4*, *FLNC*, *TUBB6* and *BICC1*.

**Table S10**. circRNA expression analysis at the gene level from RNA-seq data of 54 NSCLC cell lines. Back splicing reads per gene normalized to the sequencing library size for 12251 genes in 54 NSCLC cell lines [28, 38]. Fold change values and significance comparing fast versus slowly migrating cell lines on different coating conditions for each gene are also provided.

**Table S11**. Differentially expressed circRNAs at the gene level regulated between fast and slow cell lines. List of 28 genes obtained from RNA-seq data with differential circRNA expression selected through the filtration criteria described (Supplementary figure 11A). Expression values are provided for 18 fast and 26 slow cell lines which showed these fast or slow properties on the majority of coatings.

**Table S12**. circRNA expression analysis at the back-splice level from RNA-seq data of 54 NSCLC cell lines. Back splicing reads per individual circRNA (back-splice site) normalized to the sequencing library size for 148811 circRNAs in 54 NSCLC cell lines [28, 38]. Fold change values and significance comparing fast versus slowly migrating cell lines on different coating conditions for each circRNA are also provided.

**Table S13**. Differentially expressed circRNAs regulated between fast and slow cell lines detected at the back-splice level. List of 33 candidate circRNAs obtained from RNA-seq data and selected through the filtration criteria described (Supplementary figure 11C). Expression values provided for 18 fast and 26 slow cell lines which showed these fast or slow properties on the majority of coatings.
